# Early Inflammatory Signatures Predict Subsequent Cognition in Long-Term Virally Suppressed Women With HIV

**DOI:** 10.3389/fnint.2020.00020

**Published:** 2020-04-24

**Authors:** Leah H. Rubin, Yanxun Xu, Philip J. Norris, Xuzhi Wang, Raha Dastgheyb, Kathryn C. Fitzgerald, Sheila M. Keating, Robert C. Kaplan, Pauline M. Maki, Kathryn Anastos, Gayle Springer, Lorie Benning, Seble Kassaye, Deborah R. Gustafson, Victor G. Valcour, Dionna W. Williams

**Affiliations:** ^1^Department of Neurology, Johns Hopkins University, Baltimore, MD, United States; ^2^Department of Psychiatry, Johns Hopkins University, Baltimore, MD, United States; ^3^Department of Epidemiology, Johns Hopkins University Bloomberg School of Public Health, Baltimore, MD, United States; ^4^Department of Applied Mathematics and Statistics, Johns Hopkins University, Baltimore, MD, United States; ^5^Division of Biostatistics and Bioinformatics, Sidney Kimmel Comprehensive Cancer Center, Johns Hopkins University, Baltimore, MD, United States; ^6^Department of Laboratory Medicine, Vitalant Research Institute, University of California, San Francisco, San Francisco, CA, United States; ^7^Department of Epidemiology and Population Health, Albert Einstein College of Medicine, Bronx, NY, United States; ^8^Department of Psychiatry, University of Illinois at Chicago, Chicago, IL, United States; ^9^Department of Psychology, University of Illinois at Chicago, Chicago, IL, United States; ^10^Department of General Internal Medicine, Albert Einstein College of Medicine, Bronx, NY, United States; ^11^Department of Obstetrics and Gynecology and Women’s Health, Albert Einstein College of Medicine, Bronx, NY, United States; ^12^Department of Medicine, Georgetown University, Washington, DC, United States; ^13^Department of Neurology, SUNY Downstate Health Sciences University, Brooklyn, NY, United States; ^14^Department of Neurology, University of California, San Francisco, San Francisco, CA, United States; ^15^Department of Molecular and Comparative Pathobiology, Johns Hopkins University, Baltimore, MD, United States; ^16^Division of Clinical Pharmacology, Johns Hopkins University School of Medicine, Baltimore, MD, United States

**Keywords:** HIV, viral suppression, immune, cognition, women

## Abstract

Immunologic function is an important determinant of cognition. Here we examined the contribution of early immune signatures to cognitive performance among HIV-infected, virally suppressed women (HIV+VS) and in HIV-uninfected (HIV-) women. Specifically, we measured serum inflammatory markers, developed combinatory immune signatures, and evaluated their associations with cognition. Forty-nine HIV+VS women in the Women’s Interagency HIV Study (WIHS) who achieved viral suppression shortly after effective antiretroviral therapy (ART) initiation, and 56 matched HIV− women were selected. Forty-two serum inflammatory markers were measured within 2 years of effective ART initiation for HIV+VS women, and at an initial timepoint for HIV− women. The same inflammatory markers were also measured approximately 1, 7, and 12 years later for all women. Of the 105 women with complete immune data, 83 (34 HIV+VS, 49 HIV−) also had cognitive data available 12 years later at ≥1 time points (median = 3.1). We searched for combinatory immune signatures by adapting a dynamic matrix factorization analytic method that builds upon Tucker decomposition followed by Ingenuity^®^ Pathway Analysis to facilitate data interpretation. Seven combinatory immune signatures emerged based on the Frobenius residual. Three signatures were common between HIV+VS and HIV− women, while four signatures were unique. These inflammatory signatures predicted subsequent cognitive performance in both groups using mixed-effects modeling, but more domain-specific associations were significant in HIV+VS than HIV− women. Leukocyte influx into brain was a major contributor to cognitive function in HIV+VS women, while T cell exhaustion, inflammatory response indicative of depressive/psychiatric disorders, microglial activity, and cytokine signaling predicted both global and domain-specific performance for HIV− women. Our findings suggest that immune signatures may be useful diagnostic, prognostic, and immunotherapeutic targets predictive of subsequent cognitive performance. Importantly, they also provide insight into common and distinct inflammatory mechanisms underlying cognition in HIV− and HIV+VS women.

## Introduction

Central nervous system (CNS) complications persist during HIV infection despite effective antiretroviral therapy (ART) and impact HIV care, including ART adherence (Thames et al., 2011). A common CNS complication in the current ART era is cognitive impairment (CI). While the incidence of HIV-associated cognitive complications has markedly decreased, less severe forms of CI remain prevalent, affecting 30–60% of people with HIV (PWH) during their lifetime (Grant, 2008; Heaton et al., 2011; Cysique et al., 2014). Even in PWH who do not have dementia, CI is associated with poorer daily functioning, including financial and medication management, driving, multitasking, and vocational functioning (Heaton et al., 1996; Heaton et al., 2004; Marcotte et al., 2006; Waldrop-Valverde et al., 2010; Scott et al., 2011).

Despite the persistence and severity of CI in PWH, the underlying pathophysiology remains elusive and the underlying mechanisms are complex and multifactorial. CI has been linked to incomplete HIV suppression in the CNS despite ART (Bingham et al., 2011; Tamarit Mdel et al., 2012; Chen et al., 2014; Zayyad and Spudich, 2015; Saylor et al., 2016), compartmentalization of HIV RNA (Ritola et al., 2005), CNS escape (Joseph et al., 2018), viral rebound in the CSF (Gianella et al., 2016), viral persistence in peripheral tissues (Chun et al., 2008, 2015; Fletcher et al., 2014; Martinez-Picado and Deeks, 2016), and genetic factors leading to a persistent HIV CNS reservoir (Anuurad et al., 2009; Mavigner et al., 2009; Joska et al., 2011). Despite the heterogeneity of these mechanisms of viral persistence, immune activation and inflammation arise as a common thread that both promote and result from the CNS viral reservoir (Brouillette et al., 2016; Rubin et al., 2017; Gomez et al., 2018). However, the neuroinflammatory processes that promote CI in the context of HIV are not completely understood. While many studies have focused on individual inflammatory markers, it is unlikely that any single immune factor will serve as the sole predictor of cognition for all people. Rather, it is much more likely that a combination of multiple immune factors work in tandem to promote CI. Furthermore, it is expected that many combinatory inflammatory signatures exist that reflect unique mechanisms of CI for specific populations. To date, combinatory neuroinflammatory signatures that specifically identify CI in HIV+VS, but not HIV-uninfected (HIV-) individuals, have not been identified. As such, our goal was to identify peripheral immune signatures, in both HIV− and HIV+VS women, that were predictive of subsequent cognitive function.

Using a novel statistical analytic approach, we evaluated peripheral soluble and cellular biomarkers of immune activation and inflammation, including cytokines, chemoattractants, soluble forms of cell surface receptors, as well as growth and metabolic factors, and their prospective (median = 3.1 years post-measurement of immune markers) associations with cognitive function in a sample of women with HIV who remained virally suppressed for over a decade after initiating effective ART and a well-matched sample of HIV− women.

## Materials and Methods

### Participants

A limited number of Women’s Interagency HIV Study (WIHS) participants (Barkan et al., 1998; Bacon et al., 2005) were tested for biomarkers, based on key characteristics of interest for the WIHS. The initial selection of women was based on a nested case-control study, with the cases being women who were effective ART-initiators while on study, at least 20 HIV RNA-suppressed visits (at least 10 years), with viral suppression at the 2nd or 3rd ART visit (where each visit is approximately 6 months apart) and viral suppression at the 4th or 5th ART visit (*n* = 84). These 84 cases were matched with 84 HIV-uninfected controls using propensity scores based on race (African-American or not), HCV Ab and RNA status at study baseline visit, age (±5 years), BMI, current smoking status at index visit, and CD4 cell count at the stop of phenotype period (±150). From the 168 (84 HIV+VS; 84 HIV-), we selected 49 women with HIV who achieved and maintained viral suppression shortly after effective ART initiation (within 2 years) and a matched sample of 56 HIV- women from the WIHS. Sixteen HIV+VS cases were removed as there was more than a 4 year gap between effective ART initiation and the date of collection for the first immune visit. An additional 21 women were removed in order to maintain an approximate 1 year gap between the initial and second visit with immune data available. Of the remaining 131 women, 28 were removed for not having 4 visits with immune data.

Serum levels of 42 inflammatory markers (see below) were measured using stored samples from within 2 years of effective ART initiation and 1, 7, and 12 years later for HIV+VS women. Among HIV− women, the same serum inflammatory markers were run at an initial time point, 1, 7, and 12 years later. Of the 105 women with complete immune data, 83 women (34 HIV+VS, 49 HIV−) had prospective neuropsychological test data (median = 3.1 years post inflammatory marker assessment) available at ≥1 time points. Specifically, 17 women contributed only one visit (7 HIV+VS, 10 HIV−), 12 women contributed two visits (5 HIV+VS, 7 HIV−), 12 contributed three visits (6 HIV+VS, 6 HIV−), and 42 contributed four visits (16 HIV+VS, 26 HIV−). Of the 22 women without cognitive data, fifteen (68%) were Spanish speakers and therefore not administered the neuropsychological test battery (normed for English speakers), five (23%) refused to participate in neuropsychological testing, one (4.5%) participant died before neuropsychological testing began in the WIHS, and one (4.5%) participant was deemed unable to complete neuropsychological testing. The 83 women with prospective neuropsychological test data were similar to the 105 women in terms of sociodemographic and behavioral characteristics Supplementary Table S1).

### Multiplex Serum Analyses

All luminex kits were purchased from Millipore (Billerica, MA, United States) and ELISAs were purchased from R&D systems (Minneapolis, MN, United States). Interleukin (IL)-6, IL-10, and TNF-α were assayed using the High Sensitivity MILLIPLEX kit. Fibroblast growth factor (FGF)-2, fractalkine, GRO, IL-17, interferon induced protein (IP)-10, monocyte chemotactic protein (MCP)-1, monocyte derived chemokine (MDC), and macrophage inflammatory protein (MIP)-1α were measured using the Standard Sensitivity MILLIPLEX Map kit. CXCL13/B-cell attracting chemokine (BCA)-1, CCL27/cutaneous T-cell attracting chemokine (CTACK), SDF-1 a + b, TRAIL, MIG, ITAC/CXCL11, and MIP-3b/CCL19 were measured using the Standard Sensitivity Panel II kit. Macrophage colony stimulating factor (M-CSF), CXCL9/monokine induced by gamma (MIG), CXCL11/Interferon-inducible T cell alpha chemoattractant (I-TAC), and CXCL19/MIP-3β were measured using the Standard Sensitivity Panel III kit. Soluble (s) TNFRII, sTNFRI, sVEGFRI, sVEGFR2, sIL-2Rα, and sgp130 were measured using the Soluble Receptors kit. Adiponectin, MMP-9, MPO, sE-selectin, sICAM, sVCAM, total Plasminogen Activation Inhibitor, CRP, SAA, and SAP were measured using CVD Panel 1. KIM-1, osteopontin, and renin were measured using Kidney Toxicity Panel 1. Beta2 microglobulin, Cystatin, and Clusterin were measured using Kidney Tox Panel 2. CD14 and CD163 were measured using ELISA. Standards and samples were tested in duplicate. Beads were acquired on a Labscan analyzer (Luminex) using Bio-Plex manager 6.1 software (Bio-Rad). Values that were determined to be out of range (OOR) low were assigned 1/2 the lowest value in the data set. Values that were determined to be OOR high were assigned 2 times the highest standard. Values that were extrapolated beyond the standard curve were assigned the determined value (Supplementary Table S2). Supplementary Table S3 provides the descriptive statistics for each analyte. All immune markers were log transformed and winsorized (<1% of values changed to be equal to the highest or lowest value that was within 2.5 standard deviations of the interquartile range) to normalize distributions.

### Cognitive Outcomes

The following cognitive domains and related neuropsychological tests were used: *learning and memory*, Hopkins Verbal Learning Test-Revised (HVLT-R; learning outcomes: trial 1 and total learning; memory = delay free recall); *attention/working memory*, Letter-Number sequencing (outcomes = control and experimental conditions total correct); *executive function*, Trail Making Test Part B (mental flexibility) and Stroop Test color-word trial (behavioral inhibition) (outcomes = time to completion); *psychomotor speed*, Symbol Digit Modalities Test (outcome = total correct) and Stroop Test color-naming trial (outcome = time to completion); *fluency*, letter (outcome = total correct) and semantic (outcome = total correct); and *motor function*, Grooved Pegboard (outcome time to completion, dominant and non-dominant hand). Timed outcomes were log transformed to normalize distributions and reverse scored so that better performance equaled higher values. Demographically adjusted T-scores were calculated for each outcome and T-scores were used to create domain scores consistent with previous large-scale WIHS-wide studies (Rubin et al., 2017, 2018). For each domain, a composite T-score was derived by averaging the T-scores for domains with ≥2 outcomes. If only one test in a domain was completed, the T-score for that test was used. A global neuropsychological score was derived for individuals who had T-scores for at least 4 out of 7 cognitive domains (higher values = poorer cognition). Given that cognitive performance was relatively stable over time post-immune data, our primary outcomes were the global and domain-specific T-scores itself rather than computing rate of change (slope outcomes).

### Statistical Analyses

We searched for latent immune signatures (underlying patterns of immune markers) among HIV+VS and HIV− women by adapting a dynamic matrix factorization analytic method building upon Tucker decomposition (Supplementary Material S1). We developed this method given the small sample size per group. With 42 immune markers at four time points for 49 HIV+VS and 56 HIV− women, the developed method can adequately reduce a high dimension of markers (42 immune markers) to a small number of immune signatures per group. As shown in previous studies, when data are well conditioned with a small number of factors compared to a large number of variables (42 in our case), matrix factorization can yield reliable results for sample sizes below 50 (de Winter et al., 2009). We calculated the Frobenius residual to choose the number of signatures (Supplementary Material S2). Based on these results, we subsequently used Ingenuity^®^ Pathway Analysis (IPA^®^, Qiagen, Redwood City, CA, United States), a web-based software application, to facilitate interpretation of data derived from the dynamic matrix factorization analytic method. Gene Pathways identified for each immune profile are included as Supplementary Material (Supplementary Figures S1–S13). Names for each immune profile were given based on the markers contributing to each profile combined with IPA results. Immune profile scores derived from the dynamic matrix factorization analytic method were then used as outcomes in a subsequent series of mixed-effects regression models (MRMs). The MRMs were used to examine whether the immune profile scores were stable or changing across the time course of study (12 years). Given that changes in immune profile scores over time were stable in the overall sample but individual differences were noted, our primary predictors were the average level and variability (standard deviation) of immune profile scores (Supplementary Figure S14). The average and variability in immune profile scores were then examined in relation to cognitive performance (domain-specific and global T-scores) at a given point in time (median = 3.1 years later) using MRMs to account for the number of visits each woman contributed to the analysis. MRMs were not corrected for multiple comparisons because the goal was to examine the potential clinical significance of these associations for future larger-scale studies.

## Results

### Participants

Table 1 provides sociodemographic, behavioral, and clinical factors for HIV+VS and HIV− women. The two groups were similar in all factors except for recent heavy alcohol and marijuana use (*P*’s < 0.05). Specifically, HIV- women were more likely to be recent heavy alcohol and marijuana users than HIV+VS women. Among the HIV+VS women, the mean number of years on ART was less than 1 year (range: 0.35–6.49).

**TABLE 1:** Demographic, behavioral, and clinical characteristics at the initial visit when immune makers were assessed among HIV-uninfected (HIV-) women and virally suppressed women with HIV (HIV+VS).

Variable	HIV− (*n* = 56) *n* (%)	HIV+VS (*n* = 49) *n* (%)	*P*-value
Age, M (SD)	38.0 (9.3)	38.9 (8.5)	0.62
Years of education, M (SD)	3.9 (1.0)	3.9 (1.2)	0.75
**Race/ethnicity**			0.27
Black, non-hispanic	31 (55)	21 (43)	
Hispanic	20 (36)	19 (39)	
Other	5 (9)	9 (18)	
Annual household income ≤12,000/year	33 (59)	23 (47)	0.22
Currently employed	23 (41)	15 (31)	0.27
Clinically relevant depressive symptoms^†^	17 (30)	19 (39)	0.36
Currently smoking	22 (39)	17 (35)	0.63
**Recent use**			
Heavy alcohol	6 (11)	0 (0)	0.02
Marijuana	11 (20)	3 (6)	0.04
Crack, cocaine, and/or heroin use	5 (9)	2 (4)	0.32
Hepatitis C RNA positive	14 (25)	12 (24)	0.95
Body mass index	28.6 (6.9)	28.9 (5.9)	0.81
Hypertension	10 (18)	14 (28)	0.19
Diabetes	3 (5)	6 (12)	0.21
ART treated	–	49 (100)	
CD4 count, median (IQR) Current Nadir	–	426 (242) 212 (213)	
Effective ART duration, mean (SD), years	–	0.72 (0.42)	
ART duration, mean (SD), years		2.45 (1.35)	
Prior AIDS diagnosis	–	13 (26)	

*WRAT-3, wide range achievement test standard score; Years of Education: 1, no schooling; 2, grades 1–6; 3, grades 7–11; 4, completed high school, 5, some college, 6, completed 4 years of college, and 7, attended/completed graduate school. ^†^CES-D, Center for Epidemiological Studies Depression scale ≥16 cutoff; current, refers to within the past week; recent, refers to within 6 months of the most recent WIHS visit; heavy alcohol use reflects >7 drinks/week or ≥4 drinks in one sitting; ART, antiretroviral therapy. Variables reported as *n* (%) were analyzed with Chi–square tests. Variables reported as M (SD) were analyzed t2with independent *t*-tests. Variables reported as median/IQR were analyzed with Wilcoxon–Mann–Whitney test.*

### Identification of Combinatory Immune Signatures

A panel of immune factors, rather than individual immune markers, are the most accurate reflection of the neuroinflammatory processes that contribute to cognition in PWH (Marcotte et al., 2013; Bandera et al., 2019). However, it is unclear which inflammatory signatures are directly related to HIV, and which serve as general inflammatory processes that promote CI, but are not specific to HIV. To ascertain this, we evaluated 42 soluble plasma inflammatory markers in 49 HIV+VS women within 2 years of ART initiation, and again 1, 7, and 12 years later. Similarly, we also performed this same longitudinal analysis in 56 demographically matched HIV-women. To identify mutual and distinct inflammatory signatures that existed between HIV+VS and HIV− women, we developed a novel statistical approach by adapting a dynamic matrix factorization analytic method. Utilizing this technique, we identified seven latent immune profiles, which we termed “combinatory inflammatory signatures,” in the HIV+VS and HIV- groups. This statistical method is based on the interrelated network of inflammatory processes that occur as a result of how the immune markers cooperate together, rather than individual analytes. This was achieved, in part, by evaluating the directionality of the association between each immune marker and their respective combinatory immune signature. In conjunction with IPA analysis, we assigned categorical names to each of these seven combinatory inflammatory signatures to provide an accurate description of how the networks of soluble markers interconnected to promote neuroinflammation. As our analyses focused on how the interrelated networks of inflammatory markers cooperated together, the same categorical description could have been used despite having a non-identical composition of individual immune markers. Inflammatory gene pathways identified through IPA analysis for each combinatory immune signature are included as (Supplementary Figures S1–S13).

Using our novel statistical approach and IPA analysis, we identified three combinatory inflammatory signatures implicated in neuroinflammation that were shared between HIV- and HIV+VS women: *Immune Activation & Vascular Dysfunction* (Signature 1); *T Cell-Dependent Antiviral Response* (Signature 2); and *Neuroinflammatory Signaling* (Signature 3) (Table 2). The immune markers identified in Signature 1 (*Immune Activation & Vascular Dysfunction*), as well as the directionality of their associations within this signature, were identical between HIV- and HIV+VS women. Signature 1 was defined by the neuroinflammatory profile that existed by the interrelated network of Beta-2 microgloblin, Clusterin, Cystatin c, sCD14, and sVEGFR2, which were all negatively associated with this immune signature. In contrast, this was not the case for the remaining signatures that were in common between HIV− and HIV+VS women. Because our analyses focused on *how* the interrelated networks of inflammatory mediators cooperated together, the same categorical description was assigned to Signature 2 (*T Cell-Dependent Antiviral Response*) and Signature 3 (*Neuroinflammatory Signaling*), for HIV- and HIV+VS women despite having a different composition of individual immune markers. However, the signatures were not completely distinct. In fact, two immune markers were consistent between HIV− and HIV+VS women for Signature 2 (TRAIL/CD253 and IL-10) and Signature 3 (serum amyloid A and CRP 3).

**TABLE 2:** Strongest network of inflammatory markers contributing to Combinatory Immune Signature that were common between HIV-uninfected (HIV-) women and virally suppressed women with HIV (HIV+VS).

Combinatory immune signature	HIV-serostatus	
	HIV- (*n* = 56)	HIV+VS (*n* = 49)
1	**Immune activation & vascular dysfunction**	**Immune activation & vascular dysfunction**
	*Beta-2 microglobulin, Clusterin, Cystatin C, sCD14, sVEGFR2*	*Beta-2 microglobulin*, *Clusterin*, *Cystatin C, sCD14, sVEGFR2*
2	**T Cell-dependent antiviral response**	**T Cell-dependent antiviral response**
	TRAIL/CD253, *IL-10*, *FGF-2, Fractalkine/CX3CL1*, SDF-1a + b/CXCL12, ITAC/CXCL11	TRAIL/CD253, IL-10, BCA-1/CXCL13, 6CKINE/CCL21
3	**Neuroinflammatory signaling**	**Neuroinflammatory signaling**
	*Fractalkine/CX3CL1, FGF-2*, serum amyloid A, CRP	TRAIL/CD253, serum amyloid A, CRP, *IL-10, Adiponectin*

*Markers in italics were negatively associated with the profile; otherwise, the markers were positively associated with the profile. Our analyses focused on how the interrelated networks of inflammatory mediators cooperated together. As such, the same categorical description could be assigned for Combinatory Immune Signatures, despite having non-identical composition of individual immune markers.*

In contrast to the first three signatures, the remaining four combinatory immune signatures were unique and stratified according to HIV-serostatus. For HIV- women, each of the remaining signatures reflected distinct inflammatory processes and included: *Depressive/Psychiatric Disorder Inflammatory Response* (Signature 4^HIV–^); *Anti-Inflammatory Microglial Activity* (Signature 5^HIV–^); *Cytokine-Mediated Inflammatory Response* (Signature 6^HIV–^); and *T Cell Exhaustion* (Signature 7^HIV–^) (Table 3). This was not the case for HIV+VS women (Table 4). Notably, three of the four signatures specific to HIV+VS women were indicative of a single inflammatory process: leukocyte influx into the CNS. These signatures were assigned the categorical descriptions of *Myeloid, T Cell, and Endothelial Cell Communication* (Signature 4^HIV+VS^), *Microglial Chemokine-Mediated T cell Recruitment to Brain* (Signature 5^HIV+VS^), and *Leukocyte Recruitment to Brain* (Signature 6^HIV+VS^). While these three signatures reflected a single inflammatory process, the individual immune markers comprising the immune network were not identical. There were both conserved (IL-10, Fractalkine/CX3CL1, and ITAC/CXCL11) and unique markers (CRP, IL-17, sVEGFR2, Il-6, serum amyloid A, MIP3b/CCL19, MIG/CXCL9, and FGF-2) represented within these three immune networks. The final combinatory immune signature that was uniquely present in HIV+VS women was *Oxidative Stress* (Signature 7^HIV+VS^).

**TABLE 3:** Strongest network of inflammatory markers contributing to Combinatory Immune Signatures that were specific to HIV-uninfected (HIV−) women.

Combinatory immune signature	HIV- (*n* = 56)
4^HIV–^	**Depressive/Psychiatric disorder inflammatory response**
	*IL-10*, FGF-2, *Fractalkine/CX3CL1*, IL-6
5^HIV–^	**Anti-inflammatory microglial activity**
	*Myeloperoxidase, MIP-1*α*/CCL3, IL-6*
6^HIV–^	**Cytokine mediated inflammatory response**
	IL-6, IL-10
7^HIV–^	**T Cell exhaustion**
	*Fractalkine/CX3CL1*, IL-10, *CRP*, sVEGFR2, *serum amyloid A*

*Markers in italics were negatively associated with the profile; otherwise, the markers were positively associated with the profile.*

**TABLE 4:** Strongest network of inflammatory markers contributing to Combinatory Immune Signatures that were specific to virally suppressed women with HIV (HIV+VS).

Combinatory immune signature	HIV+VS (*n* = 49)
4^HIV+VS^	**Myeloid, T Cell, and Endothelial Cell Communication**
	*IL-10, FGF-2, Fractalkine/CX3CL1, IL-17*
5^HIV+VS^	**Microglial chemokine-mediated T Cell recruitment to brain**
	*Fractalkine/CX3CL1*, IL-10, CRP, *sVEGFR2, MIP3b/CCL19, MIG/CXCL9, ITAC/CXCL11*
6^HIV+VS^	**Leukocyte recruitment to brain**
	*IL-6, serum amyloid A, MIP3b/CCL19, MIG/CXCL9, ITAC/CXCL11, FGF-2*, Fractalkine/CX3CL1
7^HIV+VS^	**Oxidative stress**
	Myeloperoxidase, IL-6, sVEGFR1, *IL-17, IL-10*

*Markers in italics were negatively associated with the profile; otherwise, the markers were positively associated with the profile.*

### Predictive Associations Between Combinatory Immune Signatures and Cognition

We were interested in determining the contribution of peripheral immune responses implicated in neuroinflammatory processes to cognitive function. Therefore, we next evaluated the predictive abilities of the combinatory immune signatures to prospectively identify cognition in 49 HIV- and 34 HIV+VS women. To do this, we used mixed-effects regression models to identify associations between the combinatory immune signatures and global and domain-specific cognition, for both groups of women. We were particularly interested in identifying combinatory immune signatures that distinguished between HIV-related and HIV-unrelated mechanisms underlying CI. Only average (not variability) immune signatures were associated with cognition. Table 5 provides descriptive statistics for the average factor loadings for the immune signatures and average neuropsychological test performance by HIV-serostatus.

**TABLE 5:** Descriptive statistics for immune signature loadings and cognitive function for the subset of participants with neuropsychological (NP) test performance data available.

	Variable	HIV− (*n* = 49) M (SD)	HIV+VS (*n* = 34) M (SD)
**Immune signature loadings**			
1	Immune activation & vascular dysfunction	−22.5(0.4)	−22.7(0.4)
2	T Cell-dependent antiviral response	0.07 (1.0)	0.02 (1.0)
3	Neuroinflammatory signaling	0.09 (0.8)	0.07 (0.7)
4^HIV–^	Depressive/Psychiatric disorder infammatory response	0.02 (0.82)	−
5^HIV–^	Anti-inflammatory microglial activity	−0.05(0.7)	−
6^HIV–^	Cytokine mediated inflammatory response	−0.04(0.4)	−
7^HIV–^	T Cell exhaustion	−0.02(0.4)	−
4^HIV+VS^	Myeloid, T Cell, and Endothelial Cell communication	−	−0.02(0.7)
5^HIV+VS^	Microglial chemokine-mediated T Cell recruitment to brain	−	0.06 (0.6)
6^HIV+VS^	Leukocyte recruitment to brain	−	0.00 (0.5)
7^HIV+VS^	Oxidative stress	−	0.04 (0.5)
**Neuropsychological test performance**			
Global NP function		3.5 (2.1)	3.2 (1.7)
Learning		48.4 (8.9)	50.7 (9.9)
Memory		48.4 (9.9)	51.6 (9.7)
Attention/WM		50.2 (9.5)	48.1 (11.2)
Executive Function		48.9 (9.8)	48.4 (9.3)
Speed		49.6 (10.5)	50.5 (9.9)
Fluency		48.5 (9.3)	50.5 (9.6)
Motor		47.6 (12.9)	50.2 (8.9)

*M, mean; SD, standard deviation; WM, working memory; global NP function, higher values equal poorer cognition.*

**FIGURE 1:**
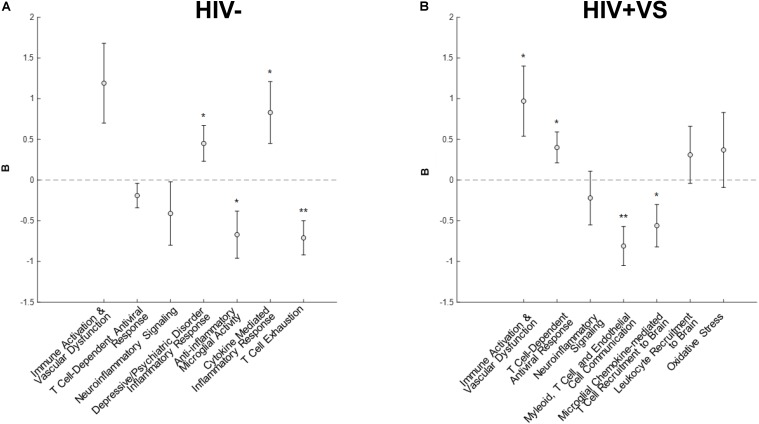
Associations between inflammatory profile scores and global cognitive performance at a subsequent point in time among **(A)** HIV- uninfected (HIV-) woman and **(B)** virally suppressed women with HIV (HIV + VS). *p < 0.05; **p < 0.01. Circles denote the unstandardized beta weights **(B)** and standard errors of **(B)**. Circles above 0 indicate positive associations whereas circles below 0 indicate negative associations. Inflammatory signatures highlighted in gray are common to both HIV– and HIV+ VS women whereas inflammatory signatures not highlighted in gray are specific to either HIV– or HIV+ VS women.

Combinatory immune signatures were equally predictive of global neuropsychological performance for both the HIV- and HIV+VS women, as four associations with global cognition were identified for both groups. Signatures 4-7^HIV–^ (Signature 4^HIV–^, *Depressive/Psychiatric Disorder Inflammatory Response*; Signature 5^HIV–^, *Anti-Inflammatory Microglial Activity*; Signature 6^HIV–^, *Cytokine-Mediated Inflammatory Response*; Signature 7^HIV–^, *T Cell Exhaustion*) were the only immune profiles significantly predicted with global cognition for HIV- women (Figure 1). That is, for HIV- women, the combinatory immune signatures that were unique to this group were significantly associated with global cognition, while those in common with HIV+VS women had no such associations. In contrast, both unique and common combinatory immune signatures were predictive of global cognition for HIV+VS women. Signatures 1^HIV+VS^ (*Immune Activation & Vascular Dysfunction*), 2^HIV+VS^ (*T Cell-Dependent Antiviral Response*), 4^HIV+VS^ (*Myeloid, T Cell, and Endothelial Cell Communication*), and 5^HIV+VS^ (*Microglial Chemokine-Mediated T cell Recruitment to Brain*) were significantly associated with global cognition for HIV+VS women. For both HIV- and HIV+VS women, some combinatory immune signatures were predictive of better global cognition (HIV-: Signatures 5^HIV–^ and 7^HIV–^; HIV+VS: Signatures 4^HIV+VS^ and 5^HIV+VS^) while other were predictive of poorer global cognition (HIV-: Signatures 4^HIV–^ and 6^HIV–^; HIV+VS: Signatures 1^HIV+VS^ and 2^HIV+VS^). Adding marijuana and heavy alcohol use to the model did not change the pattern of associations.

While our primary interest was in the combination of immune signatures, we are still able to interpret associations between each marker and cognition. To this end, we need to multiple the sign (+ or −) of the marker (W) relating to the immune profile to the sign of the beta coefficient (B) from the mixed-effects regression models. For example, the markers (W) listed in signature 1^HIV+VS^ (*Immune Activation & Vascular Dysfunction*) include five markers negatively associated with the signature (Beta-2 microglobulin, clusterin, cystatin C, sCD14, sVEGFR2). The beta coefficient (B) between the average factor loading for signature 1^HIV+VS^ and global cognition is positive. So the negative sign for the marker (−W) multiplied times the positive sign for global cognition (+B) equals a negative sign between the marker-global cognition association. Since higher scores on global cognition means poorer function, higher levels of each of these markers predict better global cognition (see Supplementary Figure S15 for an implemented example).

**FIGURE 2:**
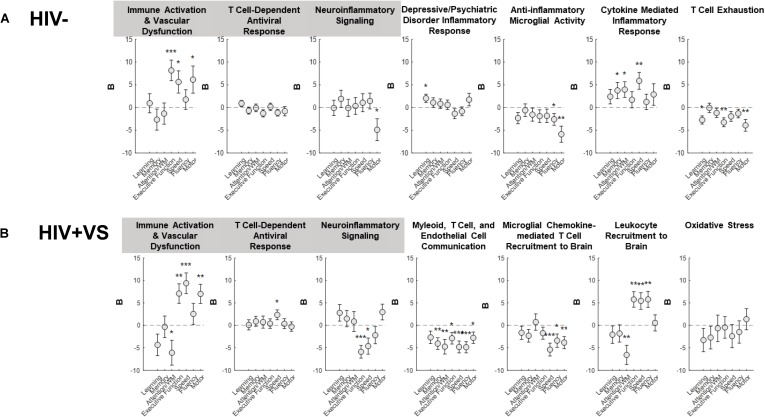
Associations between inflammatory profile scores and domain-specific cognitive performance at a subsequent point in time among **(A)** HIV- uninfected (HIV-) woman and **(B)** virally suppressed women with HIV (HIV + VS). WM, working memory; *p < 0.05; **p < 0.01; ***p < 0.001. Circles denote the unstandardized beta weights **(B)** and standard errors of **(B)**. Circles above 0 indicate positive associations whereas circles below 0 indicate negative associations. Inflammatory signatures highlighted in gray are common to both HIV– and HIV+ VS women whereas inflammatory signatures not highlighted in gray are specific to either HIV– or HIV+ VS women.

To more completely characterize the predictive abilities of the combinatory immune signatures, we also evaluated their associations with domain-specific cognition (Figure 2 and Supplementary Figure S16). In contrast to that which occurred for global cognition, the combinatory immune signatures were more predictive of domain-specific neuropsychological performance for HIV+VS women. Furthermore, the majority of combinatory immune signatures were predictive of worsening domain-level neuropsychological function in HIV+VS women. Signatures reflecting leukocyte influx into the brain (Signatures 4^HIV+VS^, 5^HIV+VS^, 6^HIV+VS^) and immune activation/vascular dysfunction (Signature 1) were highly predictive of domain-specific cognition for HIV+VS women. Each of these combinatory immune signatures were predictive of worsening speed, fluency, motor function, attention and working memory, and executive function. Notably, Myeloid, T Cell, and Endothelial Cell Communication (Signature 4^HIV+VS^) and Microglial Chemokine-Mediated T Cell Recruitment to Brain (Signature 5^HIV+VS^) were predictive of worsening cognition in 6/7 and 3/7 domains, respectively. Combinatory immune signatures reflecting T Cell-Dependent Antiviral Response (Signature 2) and Neuroinflammatory Signaling (Signature 3) were more specific, and predictive of only one and two domains: improved speed and worsening executive function and speed, respectively, in HIV+VS women. Interestingly, Immune Activation and Vascular Dysfunction (Signature 1) was predictive of improved attention/working memory, speed, and motor function, while also being predictive of worsening executive function. It is important to note that Oxidative Stress (Signature 7^HIV+VS^) was the only combinatory immune signature for HIV+VS women that was not predictive of neuropsychological function, both globally and at the domain-level. See Supplementary Figure S17 for an implemented example of marker and domain-specific cognition. Adding marijuana and heavy alcohol use to the model did not change the pattern of associations.

For HIV- women, half of the combinatory immune signatures were predictive of improved domain-level neuropsychological function, while the remainder were predictive of worsening cognition. Combinatory immune signatures reflecting Immune Activation & Vascular Dysfunction (Signature 1; executive function, speed, and motor function), Depressive/Psychiatric Disorder Inflammatory Response (Signature 4^HIV–^; learning), and Cytokine Mediated Inflammatory Response (Signature 6^HIV–^; memory, attention/working memory, and speed), were predictive of improved cognition. In contrast, Neuroinflammatory signaling (Signature 3; motor function), Anti-inflammatory Microglial Activity (Signature 5^HIV–^; fluency and motor function) and T Cell Exhaustion (Signature 7^HIV–^; learning, executive function, and motor function) were predictive of worsening domain-level cognitive function. Notably, the T Cell-Dependent Antiviral Response (Signature 2^HIV–^) was the only combinatory immune signature for HIV- women that was not predictive of neuropsychological function, both globally and at the domain-level. Adding marijuana and heavy alcohol use to the model did not change the pattern of associations.

## Discussion

We performed a prospective study to identify combinatory immune signatures indicative of neuroinflammatory processes in HIV+VS and HIV- women that predicted subsequent cognitive performance at a given point in time up to 12 years later (median ∼3 years). Forty-two immune markers were evaluated to identify combinatory immune signatures based on underlying patterns of inflammatory processes. We identified seven combinatory immune signatures: three that were common across groups (Immune Activation and Vascular Dysfunction, T Cell-Dependent Antiviral Responses, and Neuroinflammatory Signaling), while four signatures were distinct. Combinatory immune signatures were predictive of global and domain-level cognitive function for both HIV- and HIV+VS women, though differences existed between the groups. There was a greater predictive ability for the combinatory immune signatures with domain-level cognition in HIV+VS women, while the immune signatures were equally predictive of global cognitive performance for both HIV- and HIV+VS women. The predictive abilities of combinatory immune signatures were nuanced, where some were associated with worsening cognition, while others indicated improving cognitive performance. These findings are among the first to provide critical insight into the mechanisms that contribute to the heterogeneity underlying cognitive function of effective ART-treated, HIV+VS women and of demographically similar HIV- women.

Signatures reflective of *Immune Activation and Vascular Dysfunction* (Signature 1) were identical in HIV- and HIV+VS women. These mechanisms are not specific to HIV, and it is not surprising this immune signature emerged for both groups of women. Immune activation and vascular dysfunction can occur for many non-viral reasons, including metabolic disease (Jansson, 2007; Hameed et al., 2015), cardiovascular insults (Hadi et al., 2005; Frostegard, 2013), and stroke (Roquer et al., 2009; Iadecola and Anrather, 2011). Furthermore, the predictive relationship between *Immune Activation and Vascular Dysfunction* with cognition is also not surprising. Persistent immune activation may occur within the CNS and peripherally (Tchessalova et al., 2018), and is associated with CI in many psychiatric and neurological disorders (Kipnis et al., 2004; Onore et al., 2012; Krukowski et al., 2018). Vascular dysfunction is also well known to contribute to CI, which can occur through three distinct mechanisms: altered endothelial function, dysregulation of vessel tone, and altered cerebral flow (De Silva and Faraci, 2016). Each of these mechanisms affects the brain’s energy reserve and greatly contributes to cognitive decline (de Haan et al., 2006; Snyder et al., 2015; Toth et al., 2017). Similarly, the associations between the immune signature reflecting *Neuroinflammatory Signaling* (Signature 3) and cognition were expected., as the production of cytokines, innate immune and cholinergic signaling responses, and inflammatory proteins is well implicated in CI (Passos et al., 2009; Terrando et al., 2011; Griffin, 2013; Li et al., 2013; Wang et al., 2013; Peixoto et al., 2015; Reardon et al., 2018; Tchessalova et al., 2018).

Surprisingly, immune signatures implicating *T-Cell Dependent Antiviral Responses* (Signature 3) were observed in both groups of women. While an antiviral response is anticipated for the HIV+VS women, unexpectedly, this phenotype also emerged in the HIV- group. The antiviral response may have occurred in some HIV- women as a result of Hepatitis C infection. However, unlike the name suggests, antiviral responses are not restricted to viral pathogens. Instead, they may also be elicited during non-infectious insults, including metabolic disease (Li et al., 2012; Zhao et al., 2015), cancer (Zaidi and Merlino, 2011; Snell et al., 2017), cardiovascular disorders (Voloshyna et al., 2014; King et al., 2017), and autoimmune disease (Ronnblom and Eloranta, 2013; Crow et al., 2015). Therefore, the antiviral response in HIV- women may have occurred to infection with a virus other than HIV, general inflammation, or another immune stressor (Trinchieri, 2010). Over-reactive antiviral responses, particularly those associated in the Type I Interferon pathway, are associated with CI, as well as neurologic and psychiatric disease (Bonaccorso et al., 2002; Baruch et al., 2014; Sarkar et al., 2015). In PWH, a monocyte-derived type I interferon response was associated with cognitive decline (Pulliam, 2014). Our present study identified a lymphocytic, rather than myeloid, derived response that contributed to cognitive performance in only HIV+VS women.

The remaining combinatory immune signatures were specific to each group of women. Those for HIV+VS women were highly concordant with each other, where 3/4 signatures reflected a single immune response: leukocyte influx into the CNS (Signatures 4-6^HIV+VS^). As this myeloid-dependent leukocyte migration immune profile was identified in multiple clusters for HIV+VS, suggesting that this pathway is a major contributor to immune dysfunction during HIV infection. Leukocyte influx is reliably associated with viral seeding of the brain, maintaining viral reservoir in the brain, and perpetuating low level, chronic neuroinflammation during HIV infection (Nottet, 1999; Fischer-Smith et al., 2001; Grovit-Ferbas and Harris-White, 2010; Williams et al., 2014, 2015). Of note, one of the combinatory immune signatures reflective of this immune response (Signature 4^HIV+VS^) was predictive of worsening cognition in 6/7 domains, with the exception of learning, and was also significantly associated with global function. These findings indicate that sustained leukocyte infiltration into the brain occurs, despite successful viral suppression with ART, and is a major mechanism by which CI occurs for HIV+VS individuals. Oxidative stress was the other profile that occurred specifically in HIV+VS. Although oxidative stress is a well-known contributor to cognitive decline (Pratico et al., 2002; Wu et al., 2004; Droge and Schipper, 2007), for the HIV+VS women, oxidative stress was not associated with cognitive performance. While oxidative stress may contribute to other comorbidities in HIV+VS women, it does not appear to be an additional contributor to cognitive function in PWH.

We also identified a number of distinct combinatory immune signatures in HIV- women that were associated with subsequent cognitive function. Specifically, there were four immune signatures specific to HIV- women that implicated (1) *Depressive/Psychiatric Inflammatory Responses* (Signature 4^HIV–^), (2) *Anti-Inflammatory Microglial Activity* (Signature 5^HIV–^), (3) *Cytokine-Mediated Inflammatory Responses* (Signature 6^HIV–^) and (4) *T Cell Exhaustion* (Signature 7^HIV–^). Two of these were of particular interest: Signatures 4 and 7^HIV–^. Persistent CI occurs in individuals with depressive and psychiatric disorders, particularly in the domains of attention, verbal/working memory, executive function, processing speed, and visuospatial/problem solving (Gotlib and Joormann, 2010; Millan et al., 2012; Masson et al., 2016; Bernhardt et al., 2019). These CI are multifactorial and occur as a result of genetic, developmental, and environmental factors (Millan et al., 2012). We identified an inflammatory response signature associated with depressive and psychiatric disorders that predicted subsequent learning, as well as global cognition in HIV- women. There was no significant difference in the rates of clinically relevant depressive symptoms between HIV- and HIV+VS women, yet an immune profile related to the disorder was identified solely in HIV− women. This may have occurred as a result of a “masking” effect in HIV+VS women, wherein the immune response during HIV infection was driven so strongly by viral-mediated responses that it makes it difficult to assess those that are attributed to depressive and psychiatric diseases.

The identification of an immune profile related to T cell exhaustion in HIV- women was among the most interesting findings. T cell exhaustion refers to the state of dysfunction where the T cell is unable to elicit appropriate effector functions due to a transcriptional state that permits the sustained expression of inhibitory receptors. T cell exhaustion is most commonly associated with cancer and chronic infections (Wherry, 2011). Therefore, T cell exhaustion rarely occurs in a purely “healthy” population. The HIV- women involved in this study were well matched to the HIV+VS group, and while they were HIV seronegative, they had comorbidities that may contribute to a state of T cell exhaustion (Sumida et al., 2013; Massanella et al., 2015). T cell exhaustion is often implicated in CI associated with viral infections (Enose-Akahata et al., 2009; Ndhlovu et al., 2011; Grauer et al., 2015). However, the contribution of T cell exhaustion to cognitive function in the absence of pathogenic infection has not been extensively evaluated. Our finding that early plasma markers of T cell exhaustion were predictive of worsening learning, executive function, motor function and global cognition may reflect the specific comorbidities among this group of HIV- women, and may not be reflective of cognitive function in a “healthier” population of seronegative individuals. This provides insight into the importance of having a well-matched, HIV seronegative, control population when evaluating HIV and cognitive function. While it may be interesting that the T cell exhaustion cluster was not identified in HIV+VS, it is not particularly unexpected as all of the women were receiving ART and were completely virally suppressed. While not completely reversible, ART does decrease T cell exhaustion (Serrano-Villar et al., 2014). ART decreases the heightened state of immune activation, which leads to T cell exhaustion, as indicated by CD38 + HLA-DR + expression (Pallikkuth et al., 2013). Further, ART increases functionality of both CD4 and CD8 T cells, as indicated by IL-2 production upon stimulation – something which exhausted lympocytes are unable to do (Harari et al., 2004; Rehr et al., 2008).

There were a number of study limitations. This was secondary data analysis of prospective data that included a limited sample size. Furthermore, only 80% of women had subsequent neuropsychological test data available; however, the subset of women with neuropsychological test data were similar to the larger group of women in terms of sociodemographic, behavioral, and clinical characteristics. Another limitation is that comprehensive neuropsychological test data was not collected in the WIHS until 2009 thus limiting our ability to comment on the prevalence and severity of CI in our sample initially. Additionally, a targeted approach was used to evaluate the 42 immune markers. While this is a powerful methodology that allows for multiplexed analyses of pathways known to be associated with cognitive function, our results were limited as unbiased, untargeted discovery was not feasible. Additionally, interpreting directionality of the pattern of immune profile-cognition associations (e.g., higher markers, lower cognition) is difficult but possible with our analytic approach as we were interested in the combined effects of markers (signatures) rather than any one marker which has not been sufficient for understanding cognitive function. As we are the first to our knowledge to identify these exact combinatorial immune profiles, there is no precedent among immunologists as to the nomenclature of the profiles. Further, as many proteins are involved in multiple pathways, the understanding of their biological function can be context dependent (i.e., cancer vs. HIV). As such, it is difficult to ensure that our nomenclature status will satisfy all immunologists. However, there is a general consensus agreement among immunologists as to the function of the individual immune mediators that comprise the profile - which is represented in the published work of others. The nomenclature designation for the combined immune profiles was first derived in consultation with the literature to ensure we ascribed an accurate title to each group. Further, to ensure that our results were unbiased and as extensive as possible, we next used Ingenuity^®^ Pathway Analysis (IPA^®^, Qiagen, Redwood City, CA, United States), a web-based software application, to facilitate interpretation of data derived from the dynamic matrix factorization analytic method. As such, our nomenclature system is sound and strongly supported by existing ideas within the literature. Finally, our findings may not necessarily be generalizable to long-term virally suppressed men with HIV. There is a growing body of evidence demonstrating the importance of considering sex as a biological variables in studies of inflammatory biomarkers in HIV (Ticona et al., 2015; Scully, 2018; Scully et al., 2019) and in studies focusing on inflammatory contributors to cognitive function in HIV (Rubin et al., 2019). Future larger, scale studies are necessary to examine the reproducibility of these findings. Extending this work to CSF markers and brain structure and function would also further our understanding of the effects of dynamic immune signaling (peripherally and centrally) and brain health in PWH.

In sum, we identified early plasma immune signatures in HIV+VS and HIV− women that were predictive of subsequent cognitive function up to 12 years later. Importantly, our findings identified HIV-related, as well as HIV-unrelated, immune mechanisms that were associated with global and domain-specific cognitive function. Broadly, the results indicate that the underlying mechanisms that contribute to CI in these groups are both overlapping and distinct.

## Data Availability Statement

Women’s Interagency HIV Study de-identified data can be obtained by submitting a request to the Data Analysis and Coordination Center (DACC) for the Multicenter AIDS Cohort Study (MACS) and the Women’s Interagency HIV Study (WIHS) Combined Cohort Study (MACS/WIHS-CCS).

## Ethics Statement

The studies involving human participants were reviewed and approved by the Institutional Review Boards of University of Mississippi Medical Center, University of North Carolina at Chapel Hill, University of Alabama at Birmingham, University of Miami, Emory University, SUNY Downstate Medical Center, Kings County Medical Center, Montefiore Medical Center, Beth Israel Medical Center, Mount Sinai School of Medicine, Cook County Health and Hospitals System, Northwestern University, Rush University Medical Center, University of Illinois at Chicago, University of California, San Francisco, Alameda Health System, Sutter Health, Santa Clara Valley Medical Center, San Mateo Medical Center, Georgetown University, Montgomery County Department of Health and Human Services, Inova, Howard University, Whitman-Walker Clinic, University of Southern California Medical Center, Santa Barbara Neighborhood Clinics, and University of Hawai’i at Mânoa. The patients/participants provided their written informed consent to participate in this study.

## Author Contributions

LR conceived the study idea, took responsibility for the integrity of the analyses linking the immune to cognitive data, and wrote the first draft of the manuscript with DW. YX and XW took responsibility for the integrity of the statistical analyses. PN took responsibility for the Ingenuity Pathway Analyses. PN and SK conducted all of the immune analyses. All authors contributed to the writing of the manuscript and approved the final version of the manuscript.

## Conflict of Interest

The authors declare that the research was conducted in the absence of any commercial or financial relationships that could be construed as a potential conflict of interest.
