# Supplementary material for: Early Inflammatory Signatures Predict Subsequent Cognition in Long-Term Virally Suppressed Women With HIV
Source: Front Integr Neurosci. 2020 Apr 24;14:20. doi: 10.3389/fnint.2020.00020 (PMC7193823; doi:10.3389/fnint.2020.00020)
Supplement: Supplementary file 1 [file Data_Sheet_1.docx]

Supplementary Material

# Dynamic Matrix Factorization

For our problem, assume there are *N* subjects and *M* different markers. Given *K* time periods

*{t*_1_*, t*_2_*, . . . , t_K_}*, the immune markers are measured for each participant. For each time period, there is an observed matrix *X_k_* of dimension *M × N* with each element *X_k,ij_* representing the marker value of participant *i* on task *j* during time *t_k_* . We develop a dynamic matrix factorization method building upon tucker decomposition, and then classify the subjects according to patterns of change in immune markers. We will decompose *X_y_*= *W × H_k_* , here *W* is an *M × D* dimensional matrix called factor matrix, *H_k_* is an *D × N* dimensional matrix called coefficient matrix, and D represents the number of latent patterns. The basic idea is shown in Figure [1:](#_bookmark0)


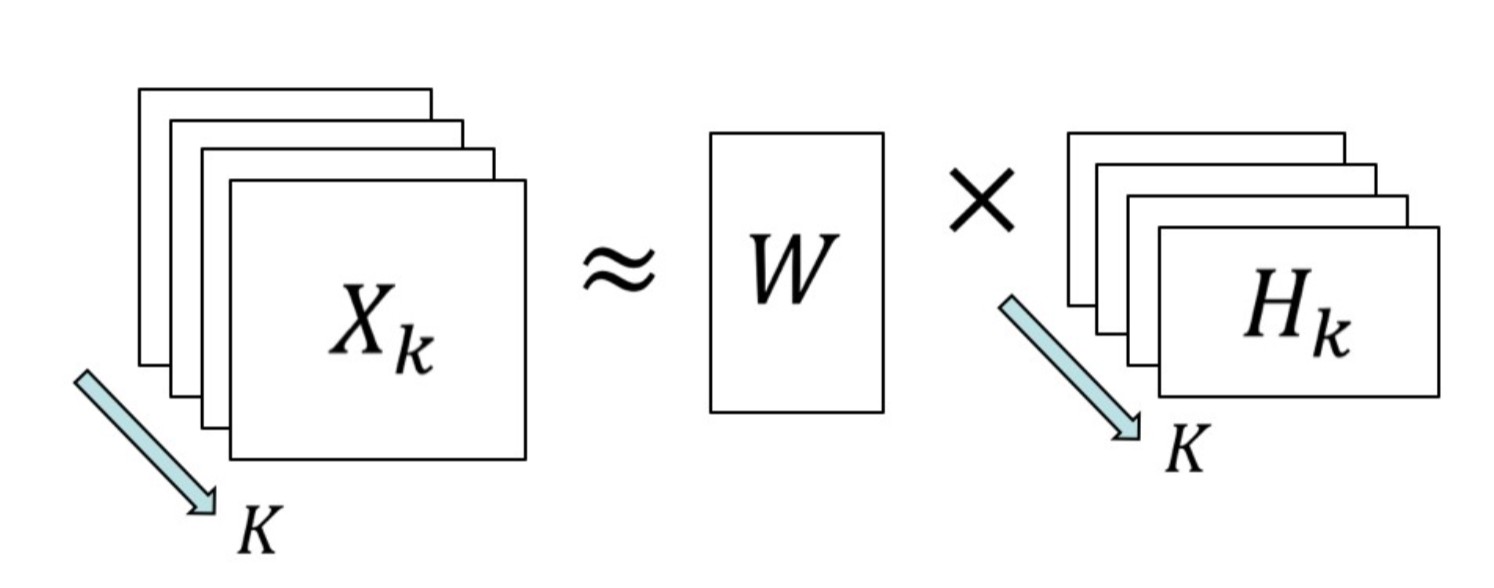


Figure 1: dynamic matrix decomposition based on tucker1

Here *W* represents the weight of each immune marker *m* in each latent immune pattern *d*, *H_k_* is the loading expression of each patient in each latent immune pattern at time *t_k_* .

## Choosing the number *D* of latent immune patterns

When the tensor is decomposed into a core tensor and factor matrix, we can calculate the residual according to

which is the Frobenius norm of the difference between the original tensor and the estimated tensor.

We ran the algorithm using different *D*, then chose the number of latent patterns as the point estimate at which an apparent decrease in the slopes of segments that connect the log value of *E* evaluated at two consecutive *D* values. We denote the log function of Frobenius log *E*(*D*) as a function of *D*, we define the second-order finite difference ∆2*logE*(*D*) by

∆2 log *E*(*D*) = 2 log *E*(*D*) *−* log *E*(*D −* 1) *−* log *E*(*D* + 1)

where *D* = *Dmin* + 1*, ..., Dmax −* 1.

# Application to Immune Marker Data

The data consists of 105 participants and 42 markers and each participants has 4 visits, which makes it a 105*42*4 tensor. Participants with HIV and HIV- participants were analyzed separately.

## Analysis of Women with HIV

### Selection of Profile number

We choose the number of profiles according to its Frobenius residual, the number is the value of K where the second-order finite difference $\Delta^{2}logE(K)$ is the largest. The second-order finite difference is illustrated in the following figure:


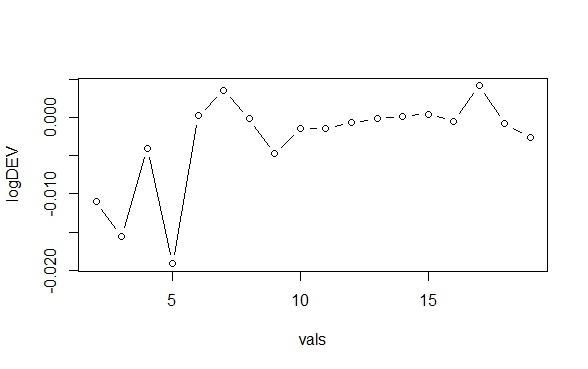


The second-order finite difference is the largest for 7 profiles.

### Factor Matrix and Its Explanation

After decomposition, we have a 49*7 factor tensor, which can be thought as the principal components on the marker mode, as for standard PCA, high values in absolute sense reflect a relevant role of the corresponding entity. The following figure is the heatmap for this factor matrix:


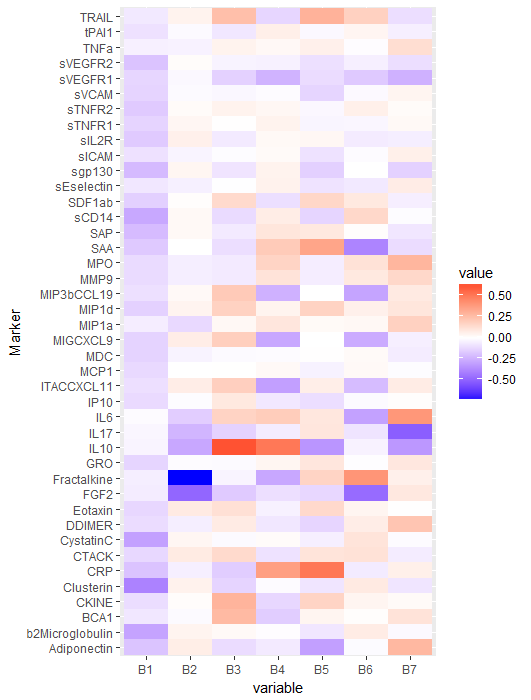


In this heatmap, color intensity correlates with the absolute value, where blue area represents negative values and red area represents positive values. Profile 1 for the marker mode mainly depends on b2Microglobulin, Clusterin, CystatinC, sCD14 and sVEGFR, all are with negative signs. For Profile 2 FGF-2, Fractalkine and IL-10 and IL-7 play important roles, all of them have negative signs like Profile 1. For Profile 3, BCA1, CKINE, IL-10, and TRAIL are important; unlike the previous two profiles, all of them have positive signs. For Profile 4, sVEGFR2, MIP3b/CCL19, MIG/CXCL9, ITAC/CXCL11 and Fractalkine are negatively related to this profile, while IL-10 and CRP are positively related to it. For Profile 5, TRAIL, SAA and CRP are positively related to this profile, while IL-10 and Adiponectin are negatively related to it. For Profile 6, SAA, MIP3b/CCL19, MIG/CXCL9, ITAC/CXCL11, IL-6 and FGF-2 are important in the negative sense, while Fractalkine is important in the positive sense. For the last profile, MPO and I-L6 play important roles with positive signs, while sVEGFR1, IL-7 and IL-10 play important roles with negative signs.

### Core Matrices Analysis with Time

The higher in absolute value an element of the core, the stronger is the interaction among the profiles involved. Since the absolute values of the core in Profile 1 are much larger and very close to each other, which means all the markers have very strong connection with Profile 1, we plot the first profile and the rest of profiles separately to analyze the pattern change over time, which can be illustrated in the following plots:


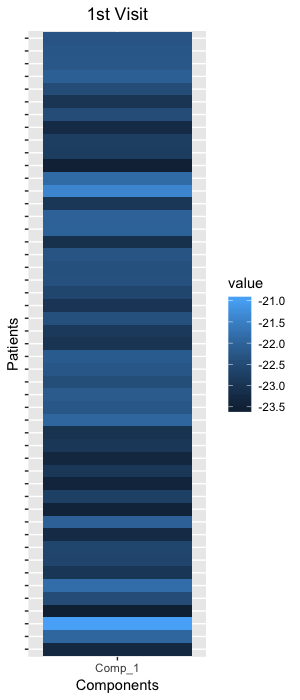

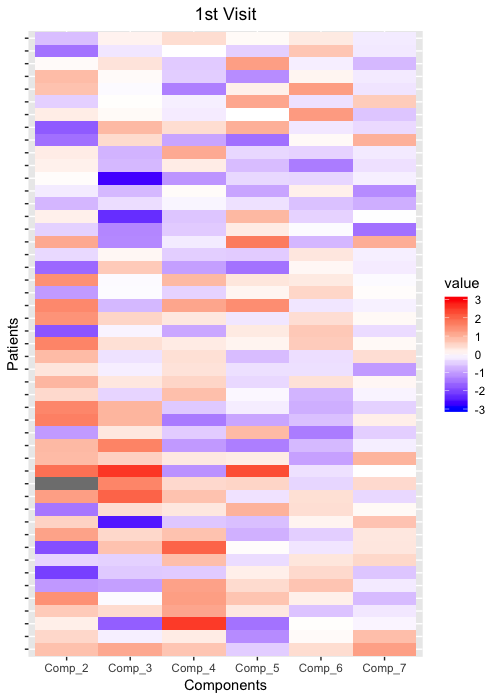


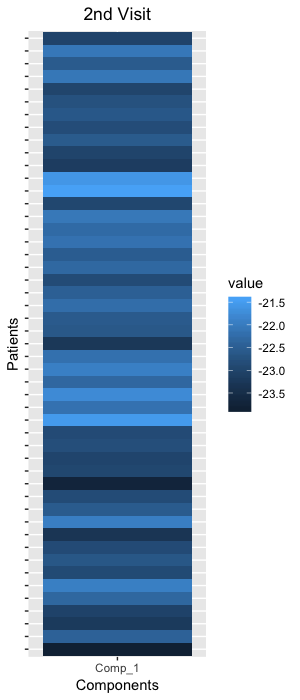

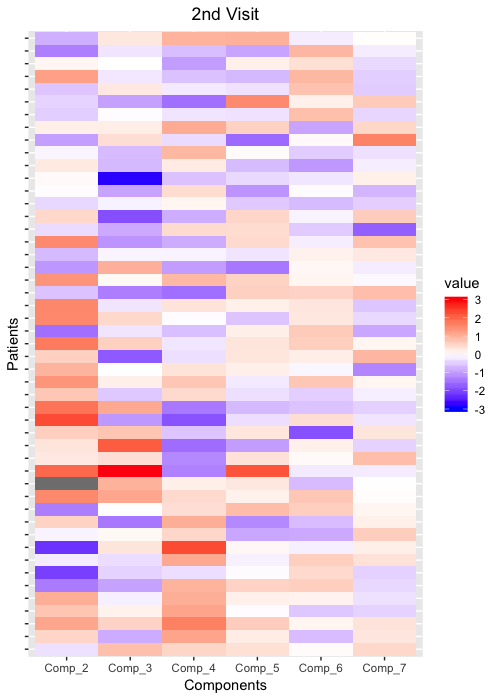


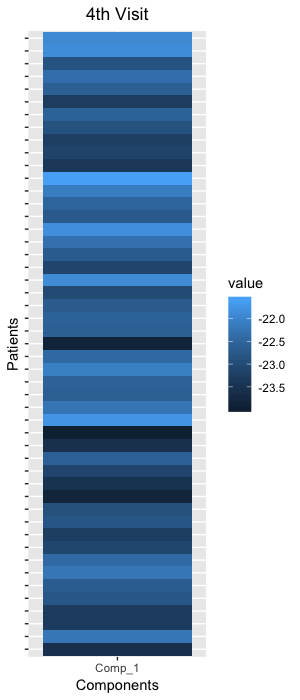

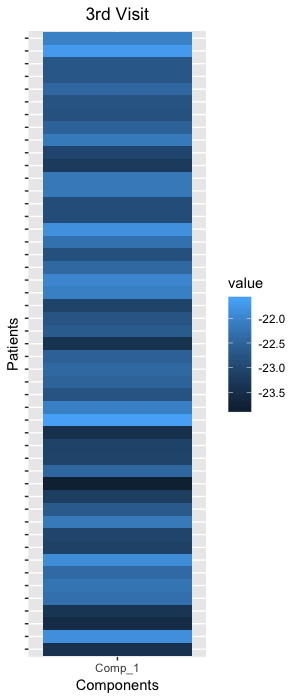

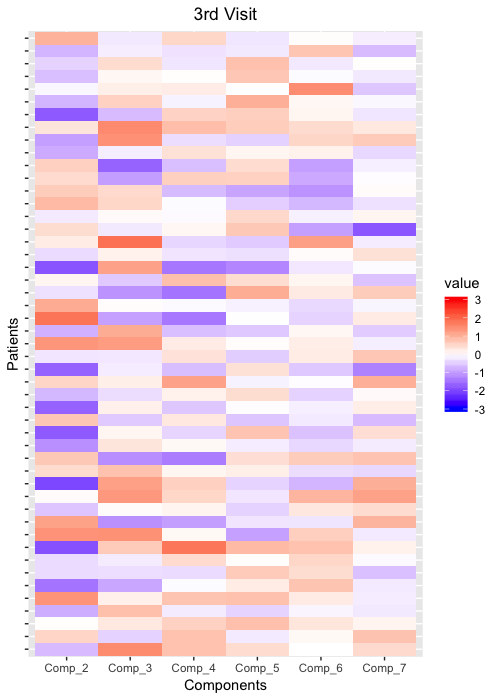


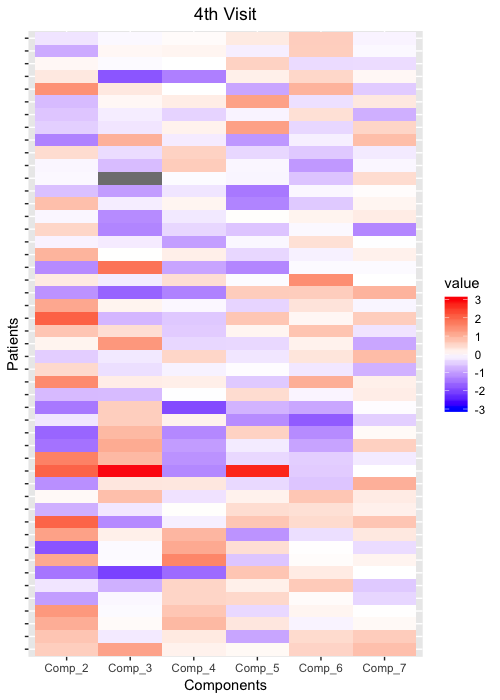


From the 1^st^ to the 2^nd^ visit, we can see there’s not much difference in the interaction between participants and profiles, only a few participants change.

From 2^nd^ to 3^rd^ visit, we can see that there are some differences between these two visits, the positive interaction between some participants in profile 2 on the 2^nd^ visit has negative interaction on the 3^rd^ visit, and for others the absolute value of interaction becomes larger with their sings unchanged, for the rest of the 5 profiles, many participants start to have stronger interaction on the 3^rd^ visit with the signs kept unchanged.

From 3^rd^ to 4^th^ visit, we can see that they’re very similar, for the second profile, the values of the participants are almost identical; for Profile 3, the values of some participants get larger such, the overall trend of the values is also getting larger; for Profile 4, some participants get weaker interaction, while some others get stronger interaction; for Profile 5, some values get larger while most of the values get smaller; for the rest of the profiles, the overall trend is getting smaller values with signs unchanged.

## Analysis of HIV-uninfected Participants

### Selection of Profile Number


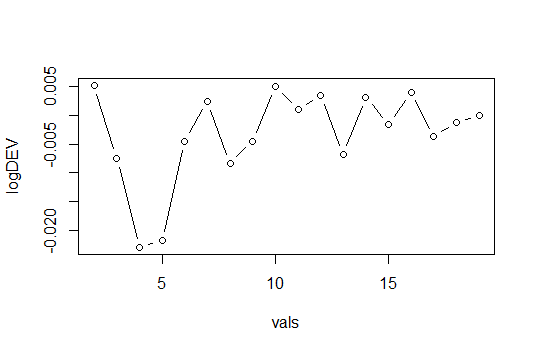
The second-order finite difference can be illustrated in the following plot:

The values of some numbers are quite similar, in order to have a proper number of profiles, we choose 7 profiles to analyze.

### Factor Matrix and Its Explanation


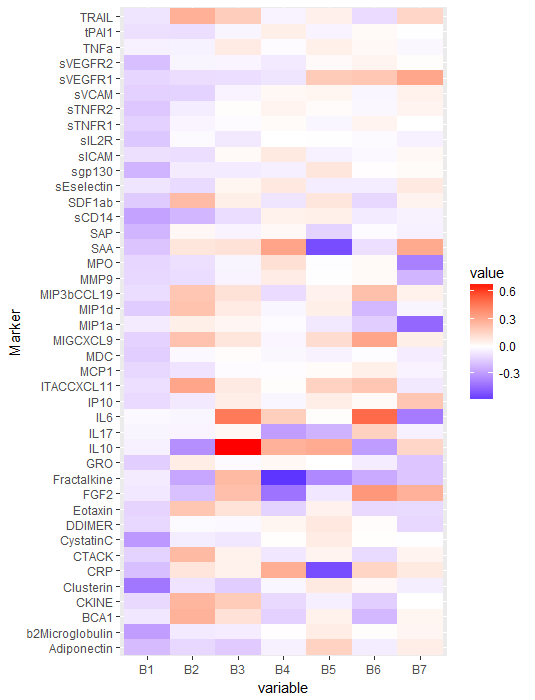


In this heatmap, darker area means higher absolute value, where blue represents negative value and red represents positive value. We see that Profile 1 for the marker mode mainly depends on b2Microglobulin, Clusterin, CystatinC, sCD14 and sVEGFR2, all are with negative signs, just like the part where participants with HIV are analyzed. For Profile 2, we can see FGF-2, Fractalkine and IL10 play important roles, both of them have negative signs, also TRAIL, SDF1ab, ITAC/CXCL11 play positive important roles. For Profile 3, IL-6 and IL-10 are important with positive signs. For Profile 4, Fractalkine and FGF2 are negatively related to this profile, while SAA and CRP are positively related to it. For Profile 5, Fractalkine, SAA and CRP are negatively related to this profile, while IL-10 and sVEGFR2 are positively related to it. For Profile 6, IL-6 and FGF-2 are important in the positive sense, while IL-10 and Fractalkine are important in the negative sense. For the last profile, MPO, MP1a, I-L6 play important roles with negative signs, which is opposite from the previous part where participants are with HIV, while sVEGFR1, SAA, IP10 and FGF-2 play important roles with positive signs.

### Core Matrices Analysis with Time

Like the previous part, since the absolute values of the core in Profile 1 are much larger and very close to each other, we’ll plot the first profile and the rest of the profiles separately to analyze the pattern change over time, which can be illustrated in the following plots:


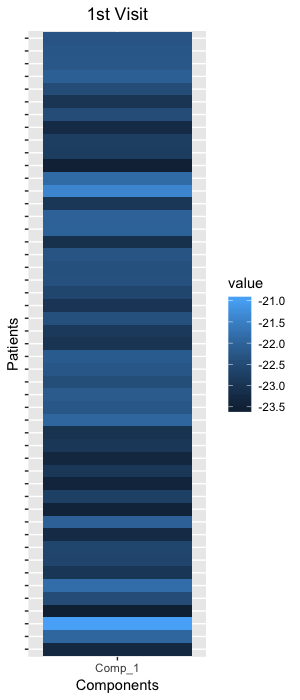

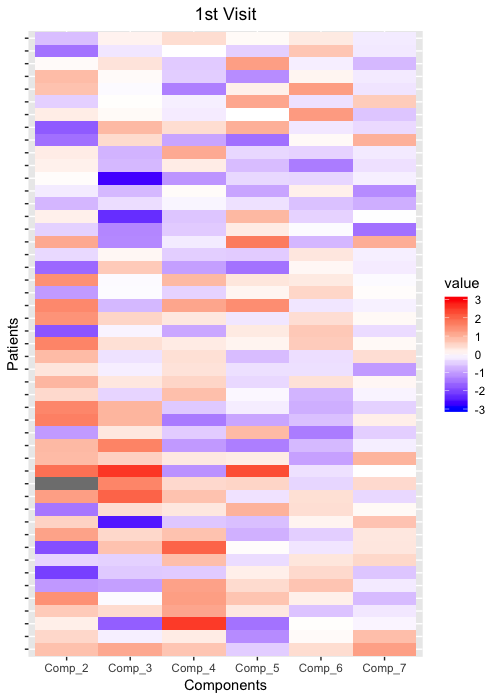


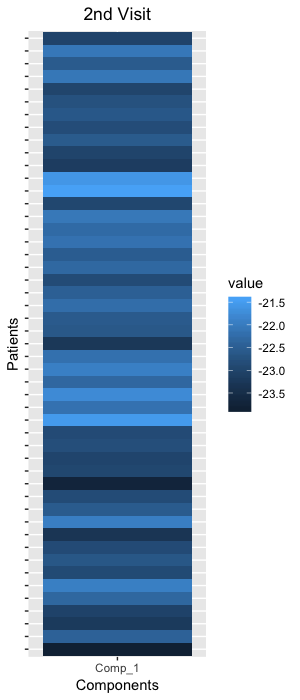

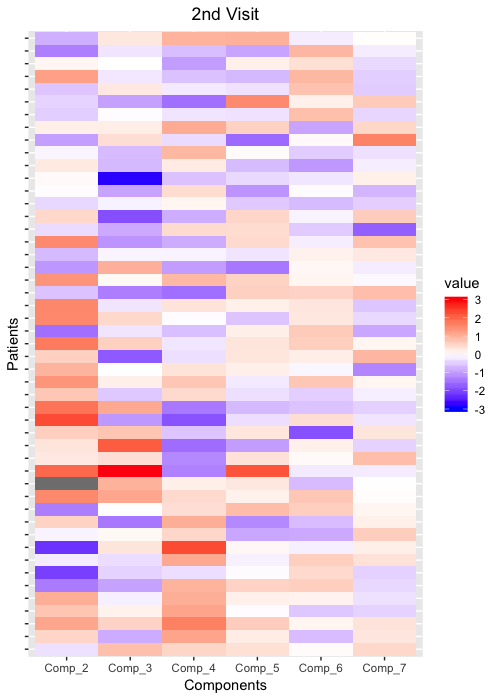


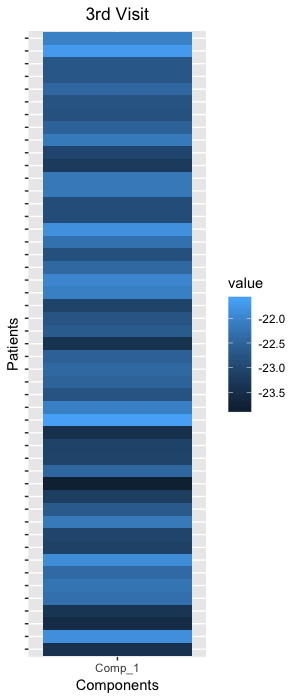

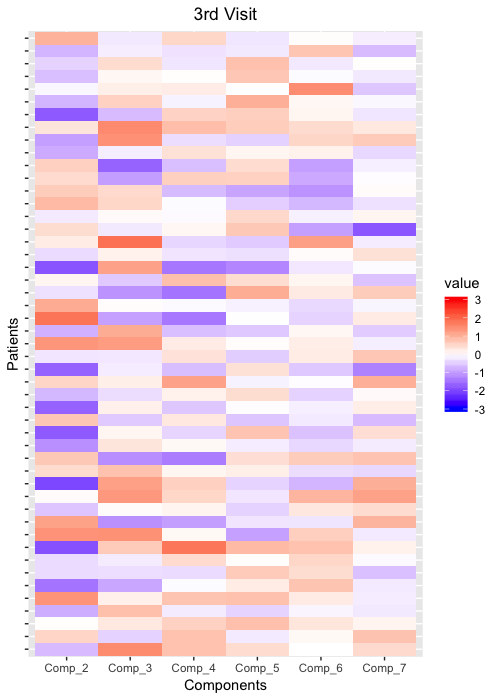


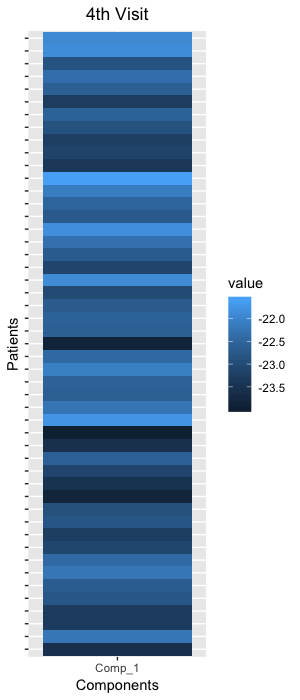

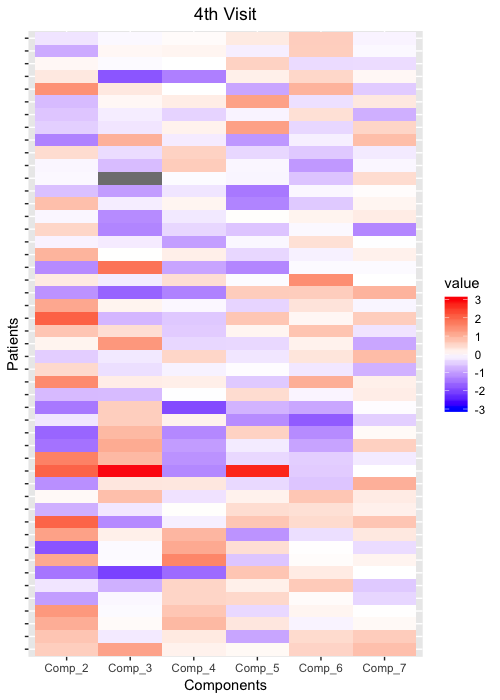


We can see that from the 1^st^ to the 2^nd^ visit, for Profile 2, there’s not much difference, most of the participants on the second visit have almost the same values as on the first visit. For the 3^rd^ profile, most of the participants maintain the same pattern, a few subject changes from positive value to negative value. For the rest of the profiles, the patterns are similar from 1^st^ visit to 2^nd^ visit.

From the second to the third visit, we can see there’s much difference. For profile 2, the values of most participants turn smaller, which means the interaction between these participants and profile 2 become less strong. Like the second profile, most of the values in the 3^rd^ profile on the 3^rd^ visit becomes smaller, and a few changes from negative to positive. For Profiles 4, 5 and 6, we can see that the trend of getting less related in the previous profiles also applies to them.

From the third visit to the fourth visit, for Profile 2, we can see that most of the values become larger. For the rest of the profiles, the same pattern of having stronger interaction applies to them.
