# Supplementary material for: Early Inflammatory Signatures Predict Subsequent Cognition in Long-Term Virally Suppressed Women With HIV
Source: Front Integr Neurosci. 2020 Apr 24;14:20. doi: 10.3389/fnint.2020.00020 (PMC7193823; doi:10.3389/fnint.2020.00020)

**Supplemental Figure 1**. Pathway analysis of the gene network associated with Immune Signature 1 for HIV- and HIV+VS women. Inflammatory markers contributing to the latent signature of the factor matrix are denoted by filled shading.

**
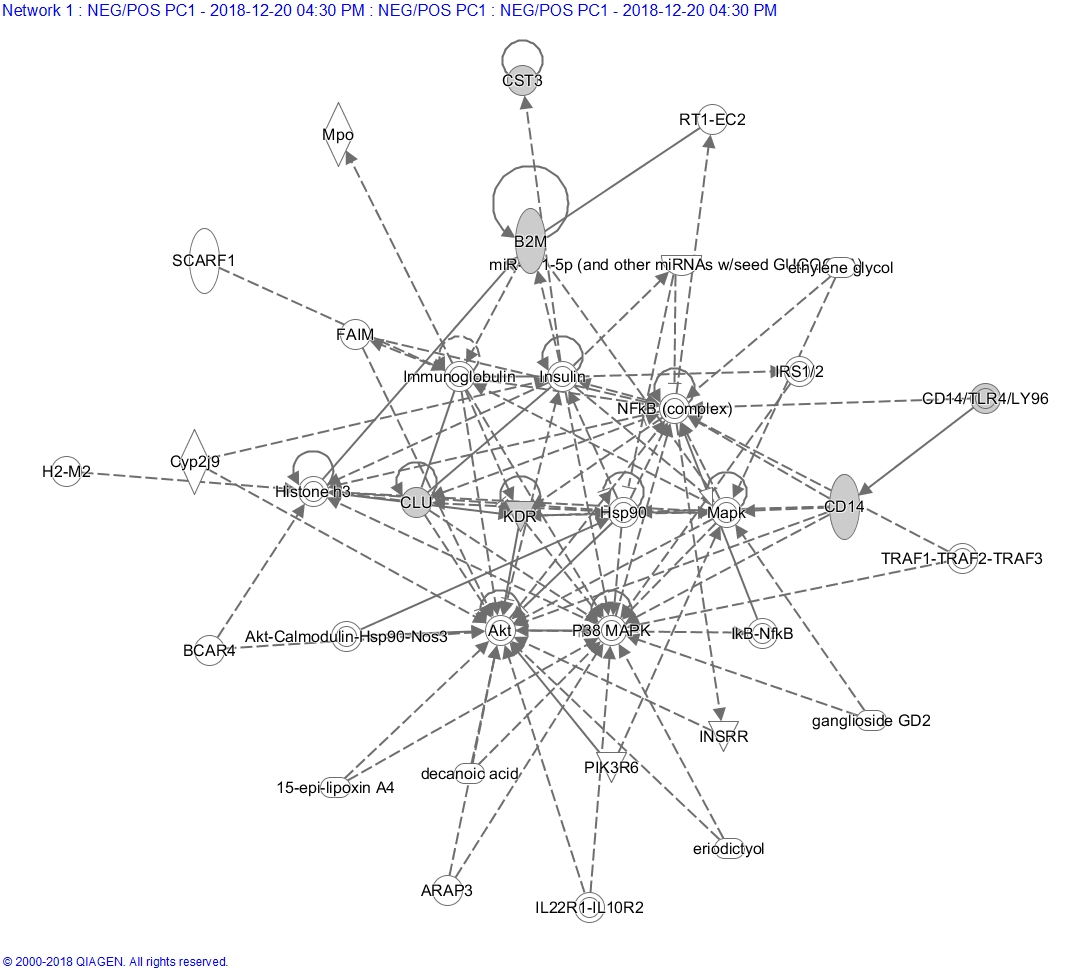
**

**Supplemental Figure 2**. Pathway analysis of the gene network associated with Immune Signature 2 for HIV- and HIV+VS women. Inflammatory markers contributing to the latent signature of the factor matrix are denoted by filled shading.

**
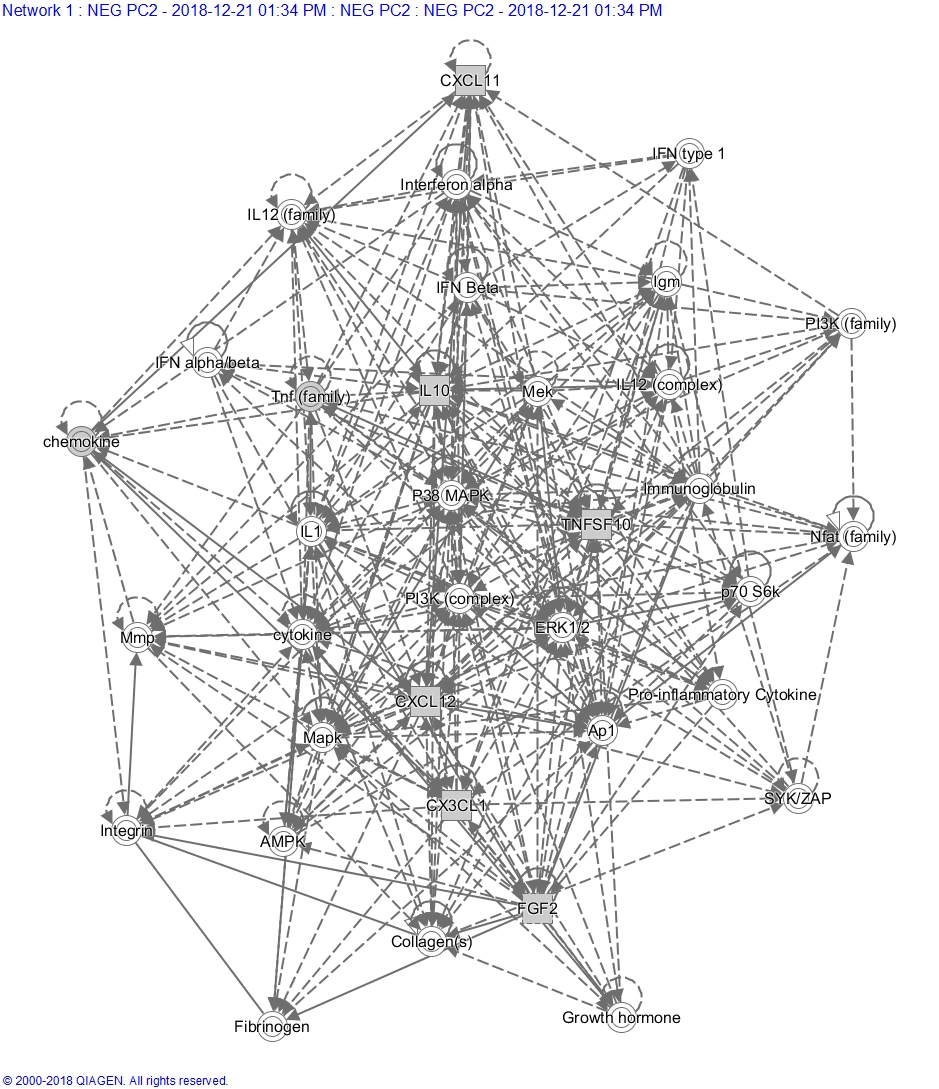
**

**Supplemental Figure 3**. Pathway analysis of the gene network associated with Immune Signature 3 for HIV- women. Inflammatory markers contributing to the latent signature of the factor matrix are denoted by filled shading.

**
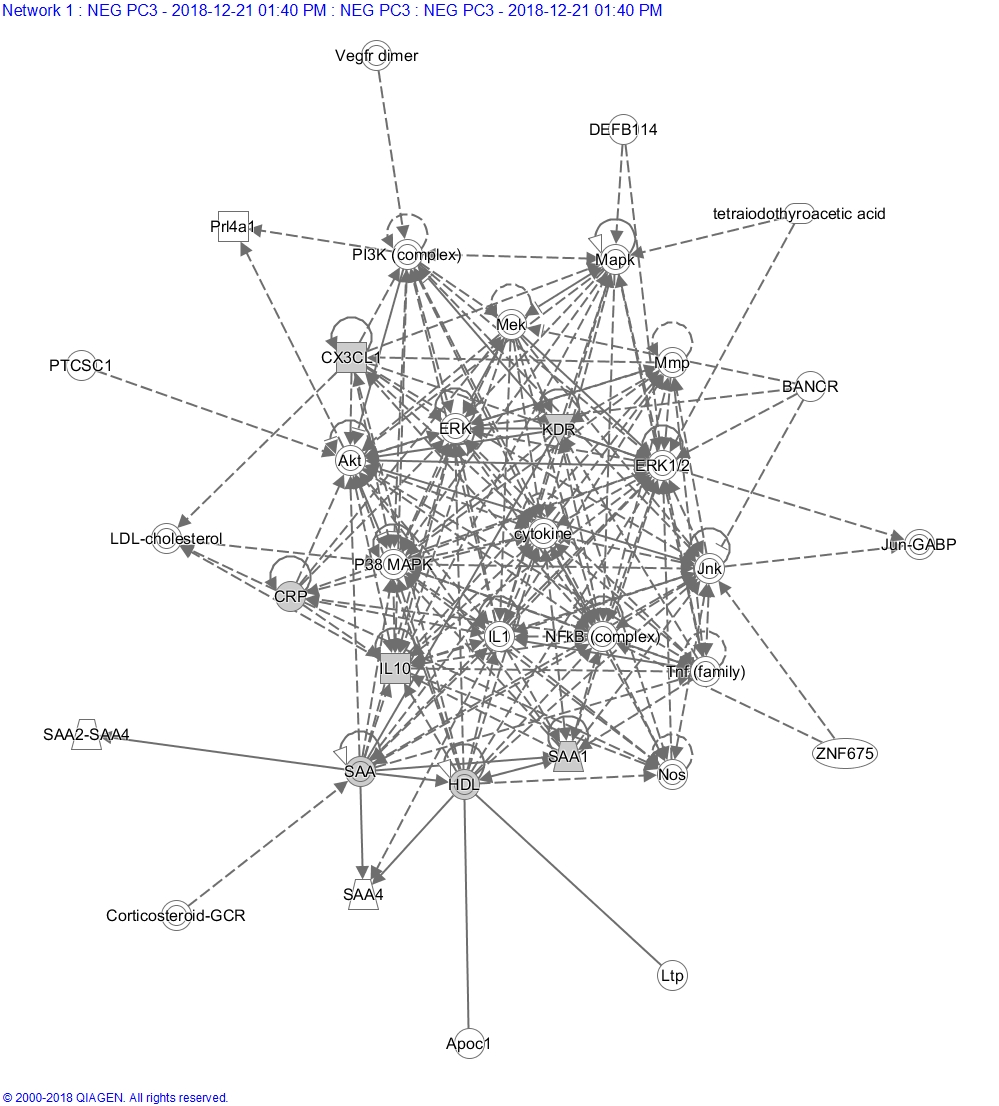
**

**Supplemental Figure 4**. Pathway analysis of the gene network associated with Immune Signature 4 for HIV- women. Inflammatory markers contributing to the latent signature of the factor matrix are denoted by filled shading.

**
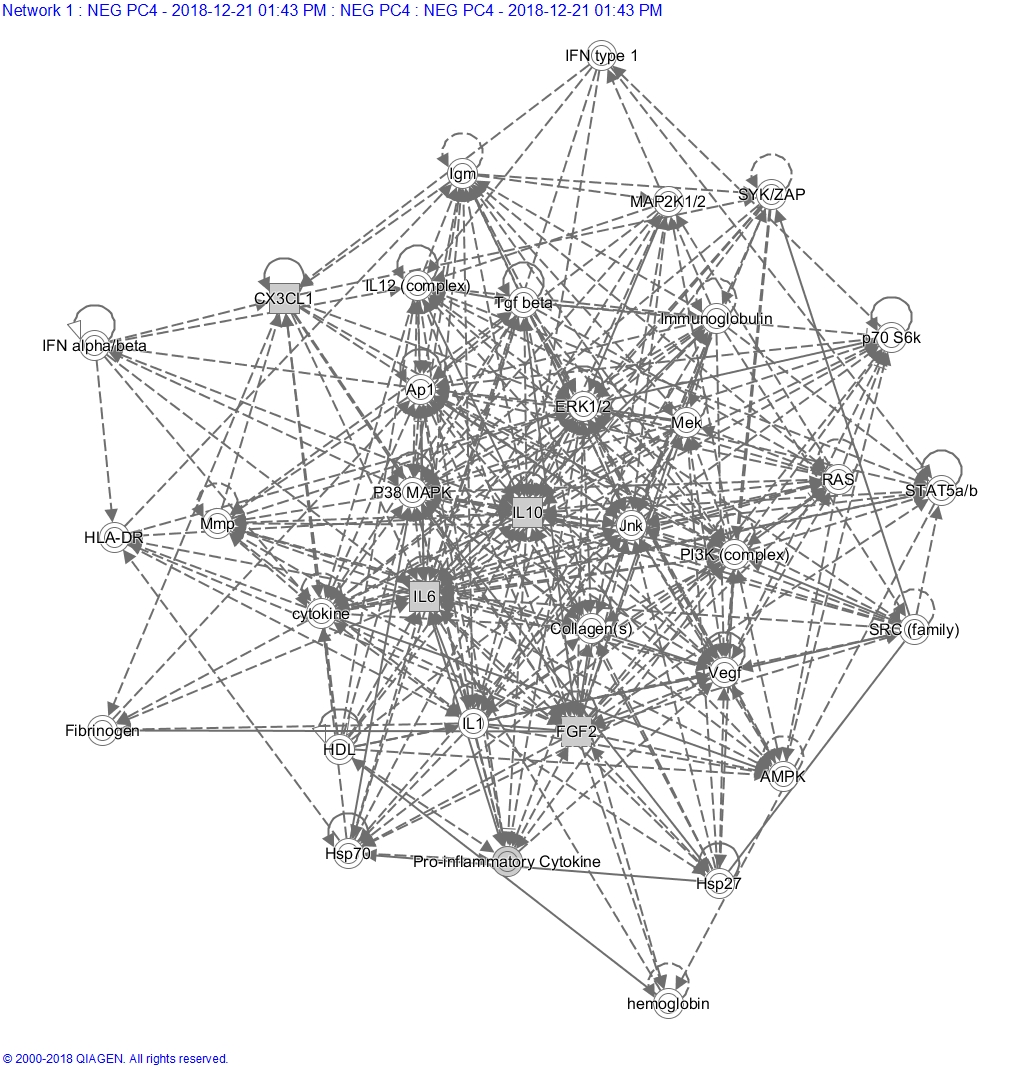
**

**Supplemental Figure 5**. Pathway analysis of the gene network associated with Immune Signature 5 for HIV- women. Inflammatory markers contributing to the latent signature of the factor matrix are denoted by filled shading.

**
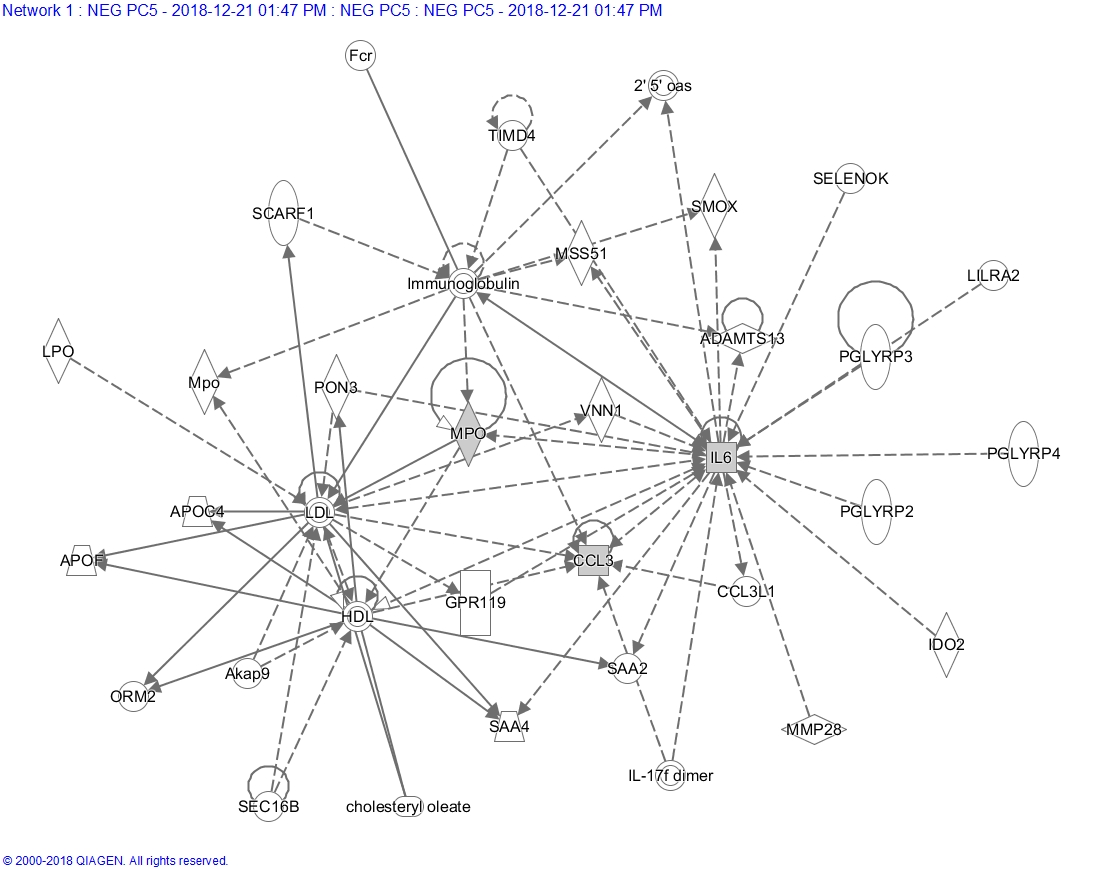
**

**Supplemental Figure 6**. Pathway analysis of the gene network associated with Immune Signature 6 for HIV- women. Inflammatory markers contributing to the latent signature of the factor matrix are denoted by filled shading.

**
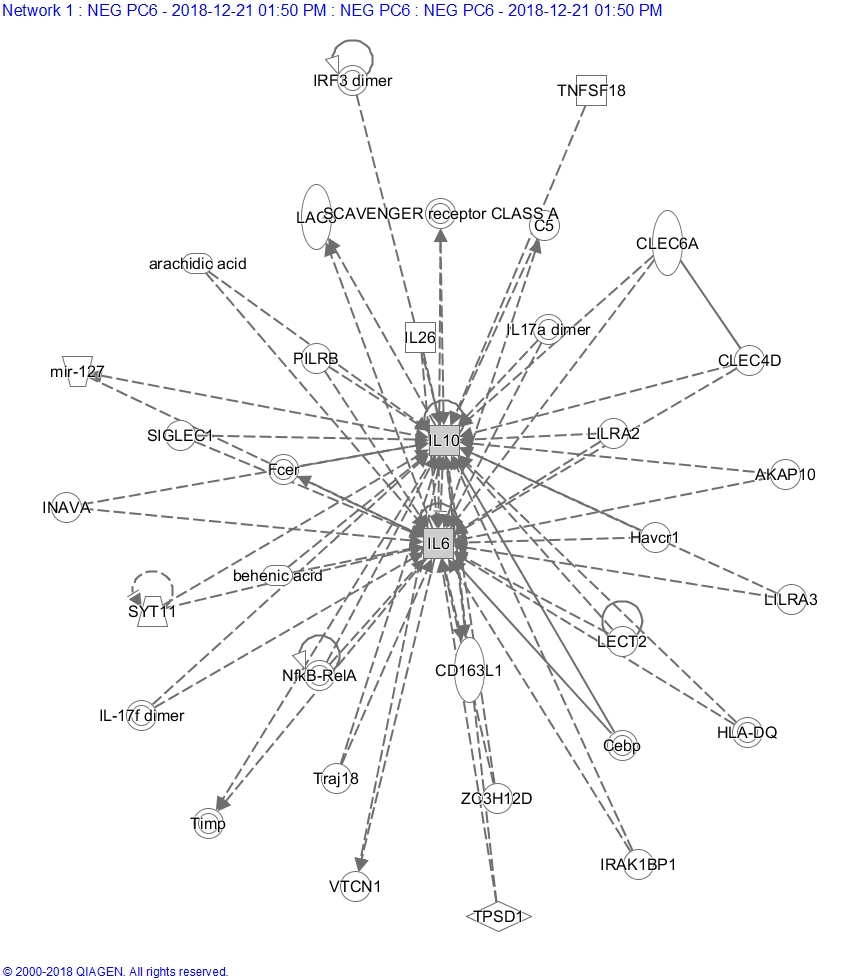
**

**Supplemental Figure 7**. Pathway analysis of the gene network associated with Immune Signature 7 for HIV- women. Inflammatory markers contributing to the latent signature of the factor matrix are denoted by filled shading.

**
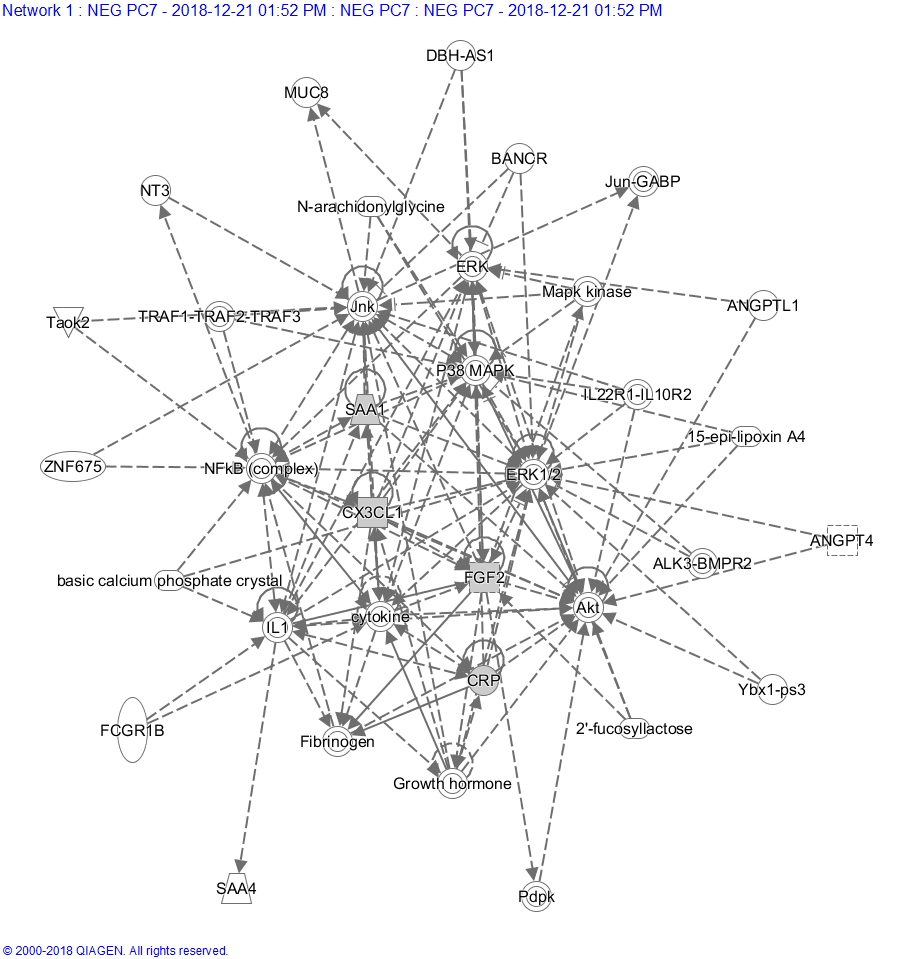
**

**Supplemental Figure 8**. Pathway analysis of the gene network associated with Immune Signature 2 for HIV+VS women. Inflammatory markers contributing to the latent Signature of the factor matrix are denoted by filled shading.

**
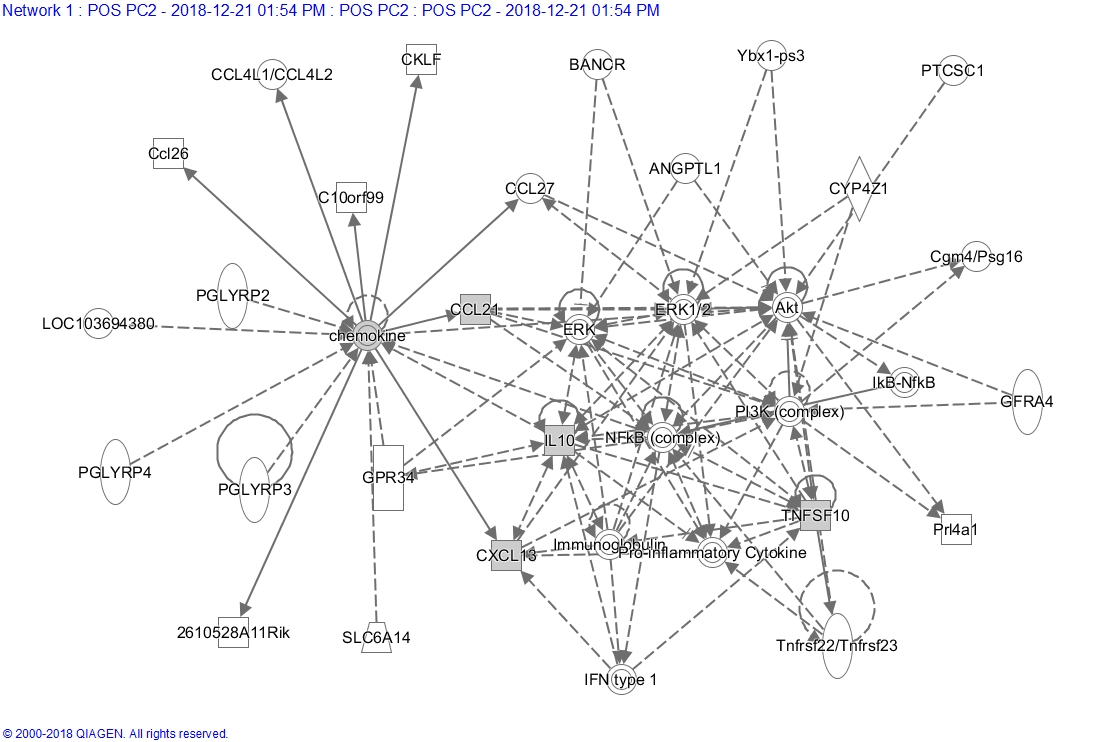
**

**Supplemental Figure 9**. Pathway analysis of the gene network associated with Immune Signature 3 for HIV+VS women. Inflammatory markers contributing to the latent signature of the factor matrix are denoted by filled shading.

**
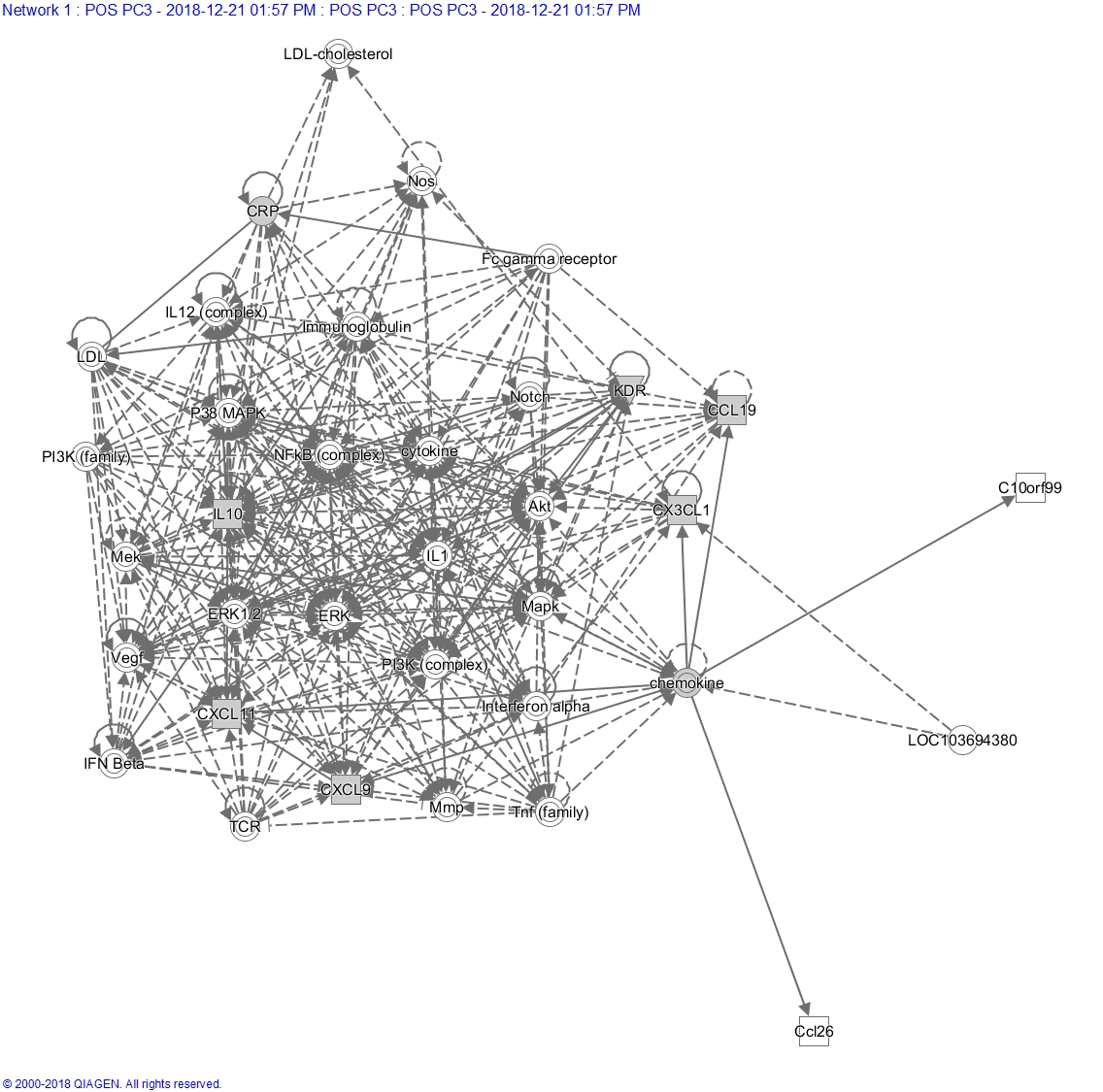
**

**Supplemental Figure 10**. Pathway analysis of the gene network associated with Immune Signature 4 for HIV+VS women. Inflammatory markers contributing to the latent signature of the factor matrix are denoted by filled shading.

**
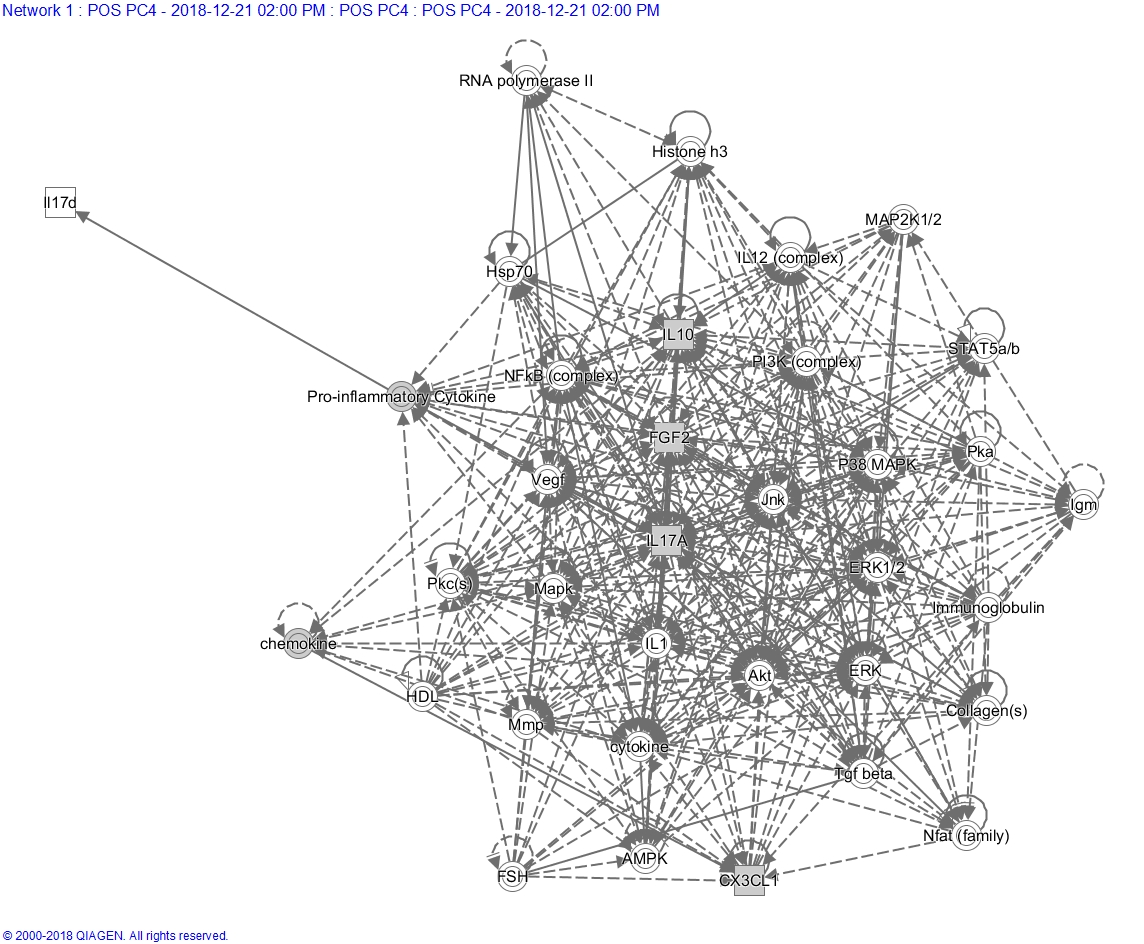
**

**Supplemental Figure 11.** Pathway analysis of the gene network associated with Immune Signature 5 for HIV+VS women. Inflammatory markers contributing to the latent signature of the factor matrix are denoted by filled shading.

**
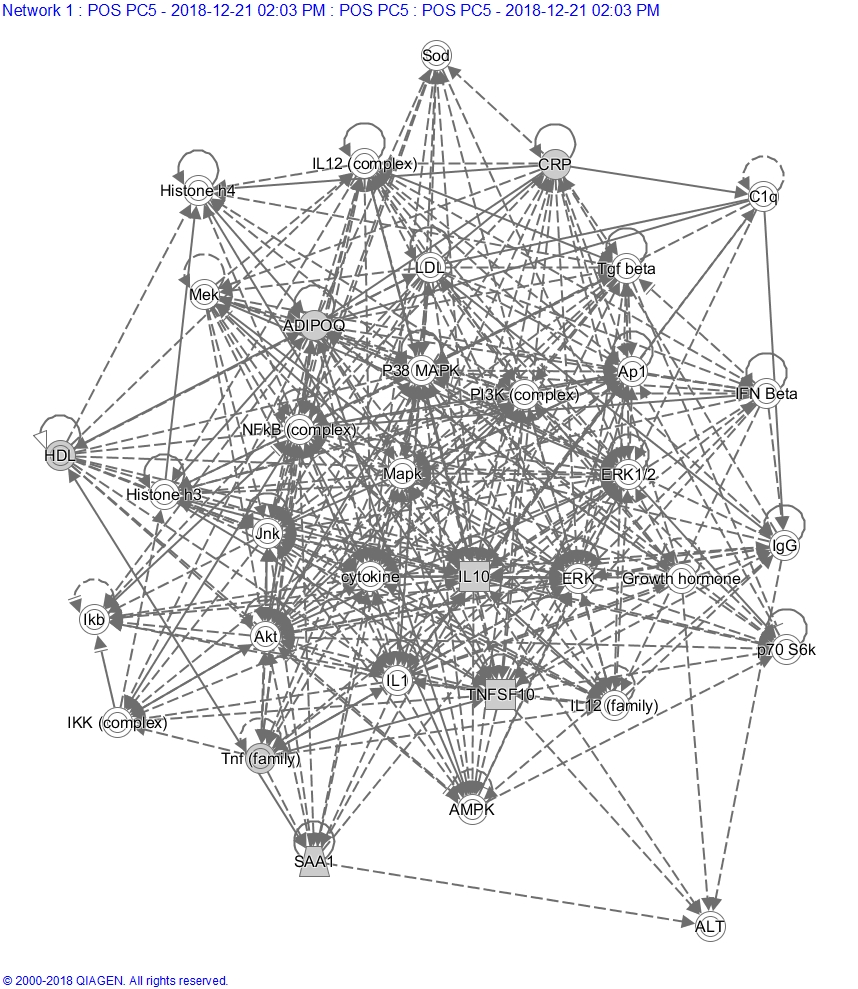
**

**Supplemental Figure 12**. Pathway analysis of the gene network associated with Immune Signature 6 for HIV+VS women. Inflammatory markers contributing to the latent signature of the factor matrix are denoted by filled shading.

**
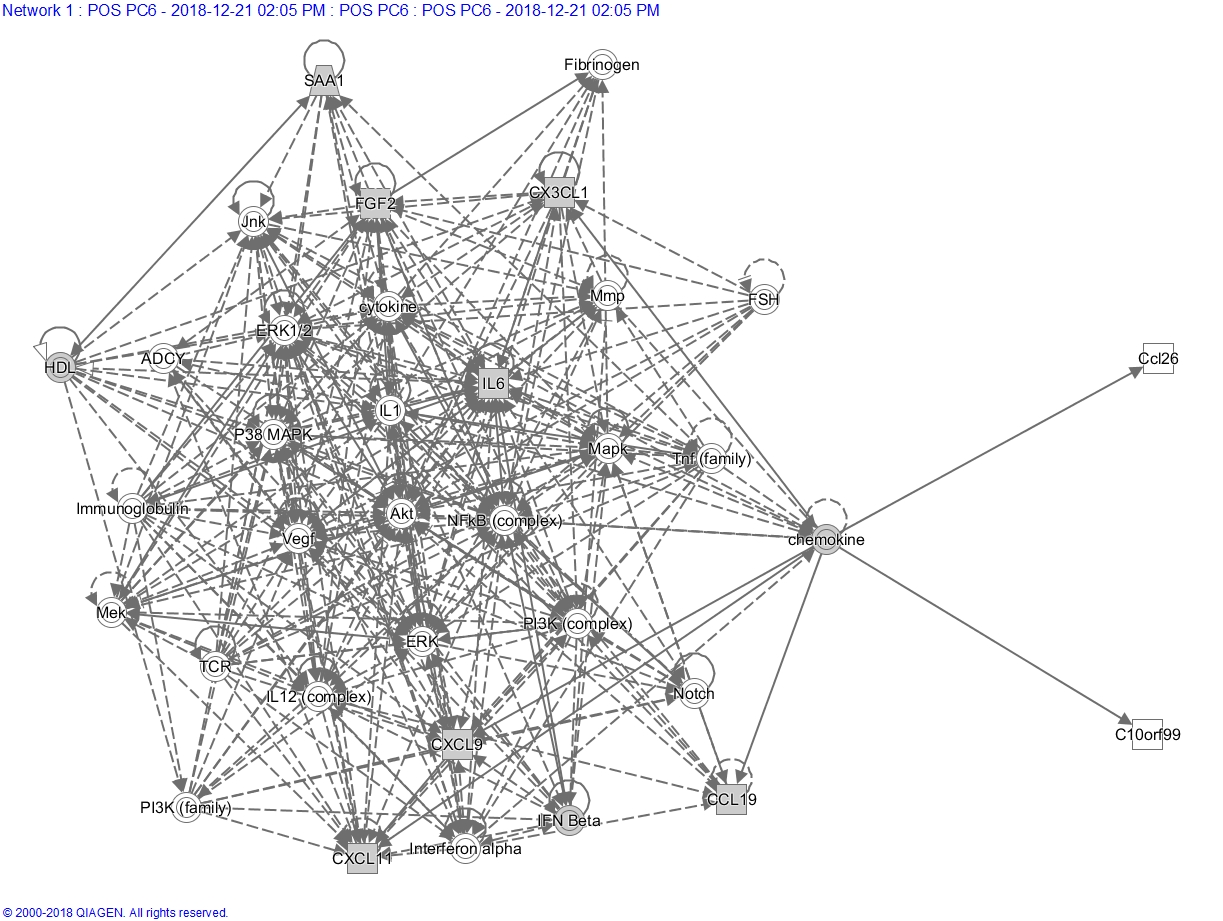
**

**Supplemental Figure 13.** Pathway analysis of the gene network associated with Immune Signature 7 for HIV+VS women. Inflammatory markers contributing to the latent signature of the factor matrix are denoted by filled shading.

**
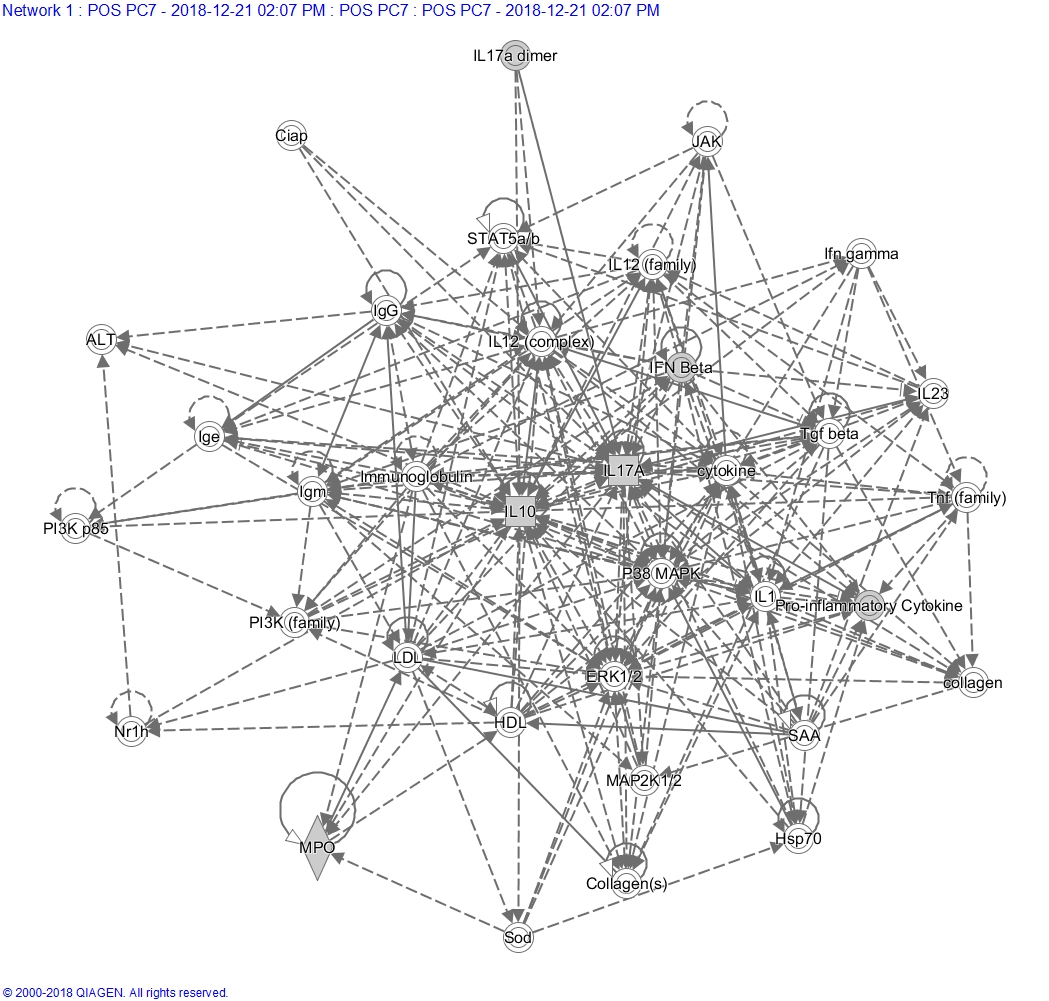
**

**Supplemental Figure 14.** Inflammatory profile scores over time (initial, 1 year, 7 years, and 12 years) among (A) HIV-uninfected (HIV-) women and (B) virally suppressed women with HIV (HIV+VS).

**Supplemental Figure 15.** Example of an application to better understand marker-global cognition associations. We applied the 1^st^ combinatory immune signature among HIV+VS women.

**
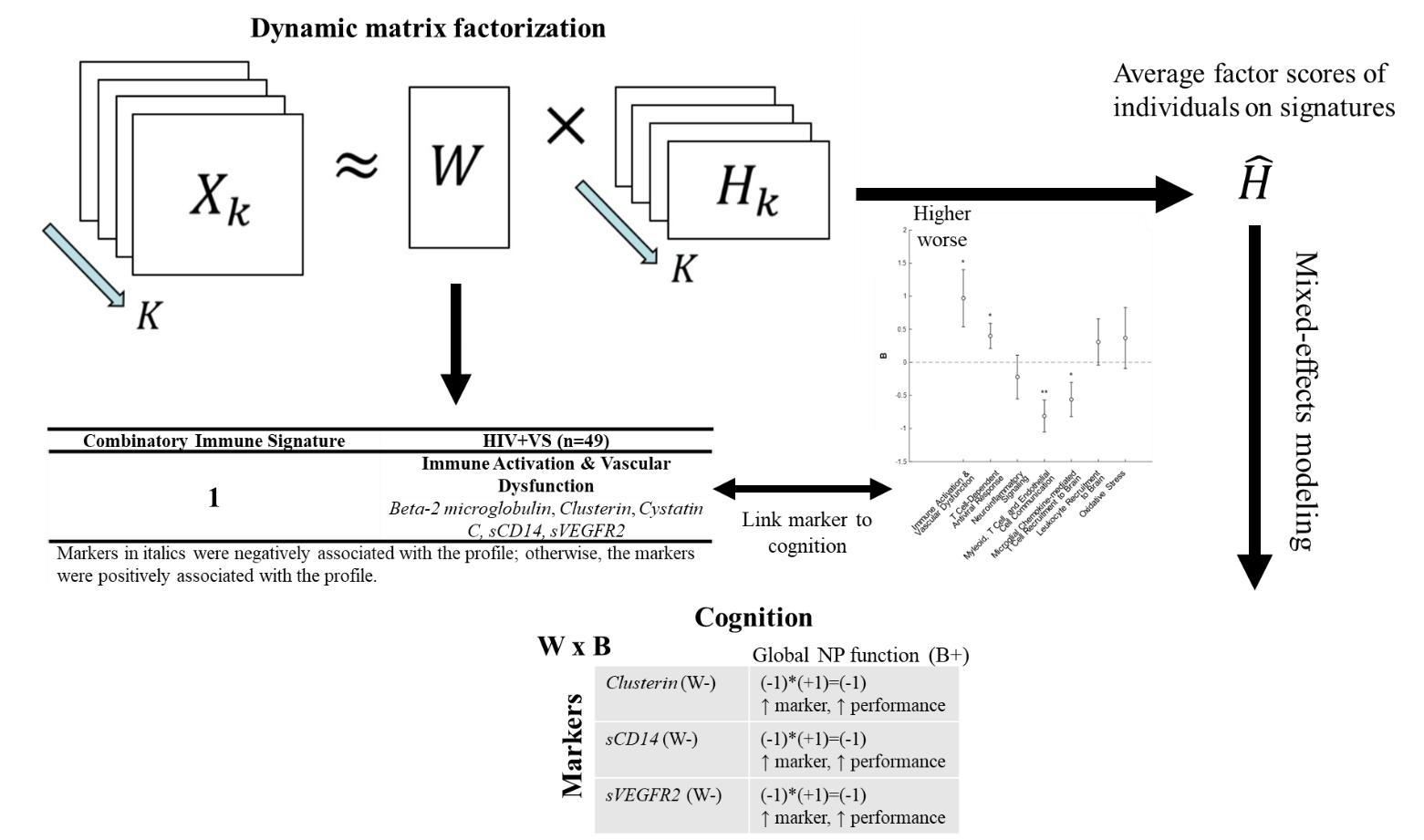
**

**Supplemental Figure 16**. Pearson correlations between mean inflammatory profile scores and cognitive performance at a point in time among (A) HIV-uninfected (HIV-) women and (B) virally suppressed women with HIV (HIV+VS).


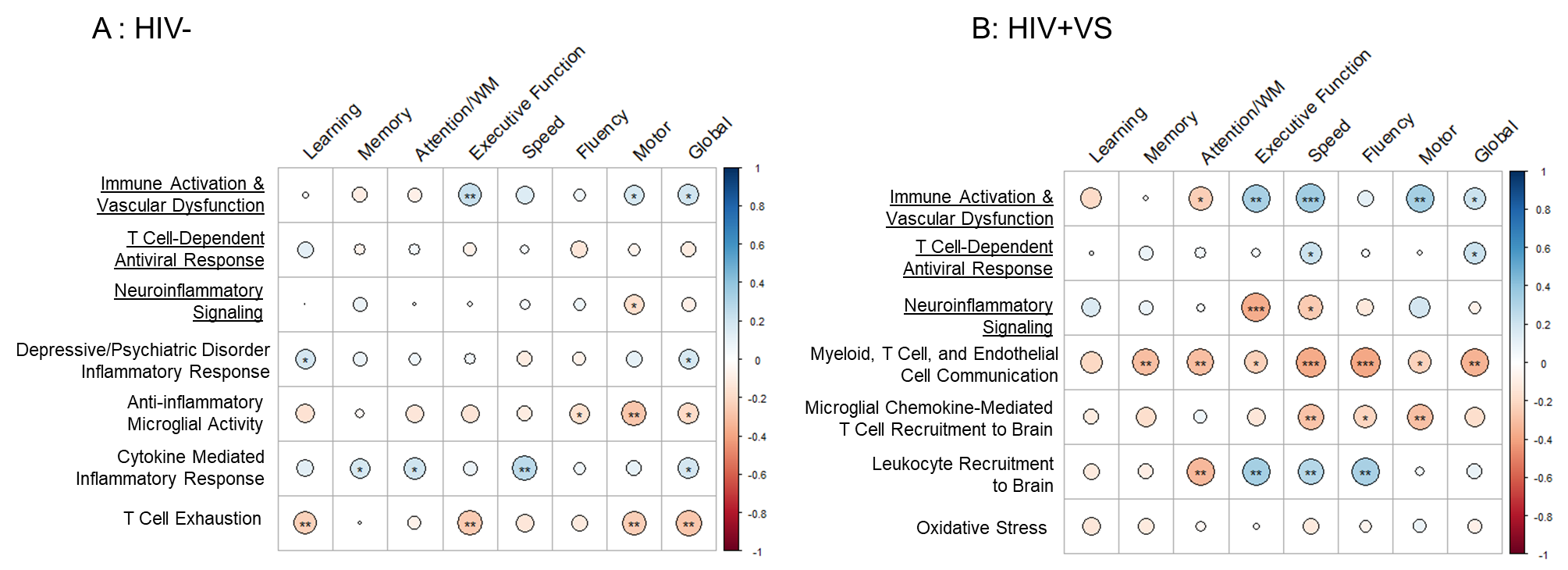


Note. WM=working memory; *p<0.05; **p<0.01; ***p<0.001. Underlined inflammatory profiles are common to both HIV- and HIV+VS women whereas no underlining indicates inflammatory profiles specific to either HIV- or HIV+VS women.

**Supplemental Figure 17**. Example of an application to better understand marker-domain-specific cognition associations. We applied the 6^st^ combinatory immune signature among HIV+VS women.


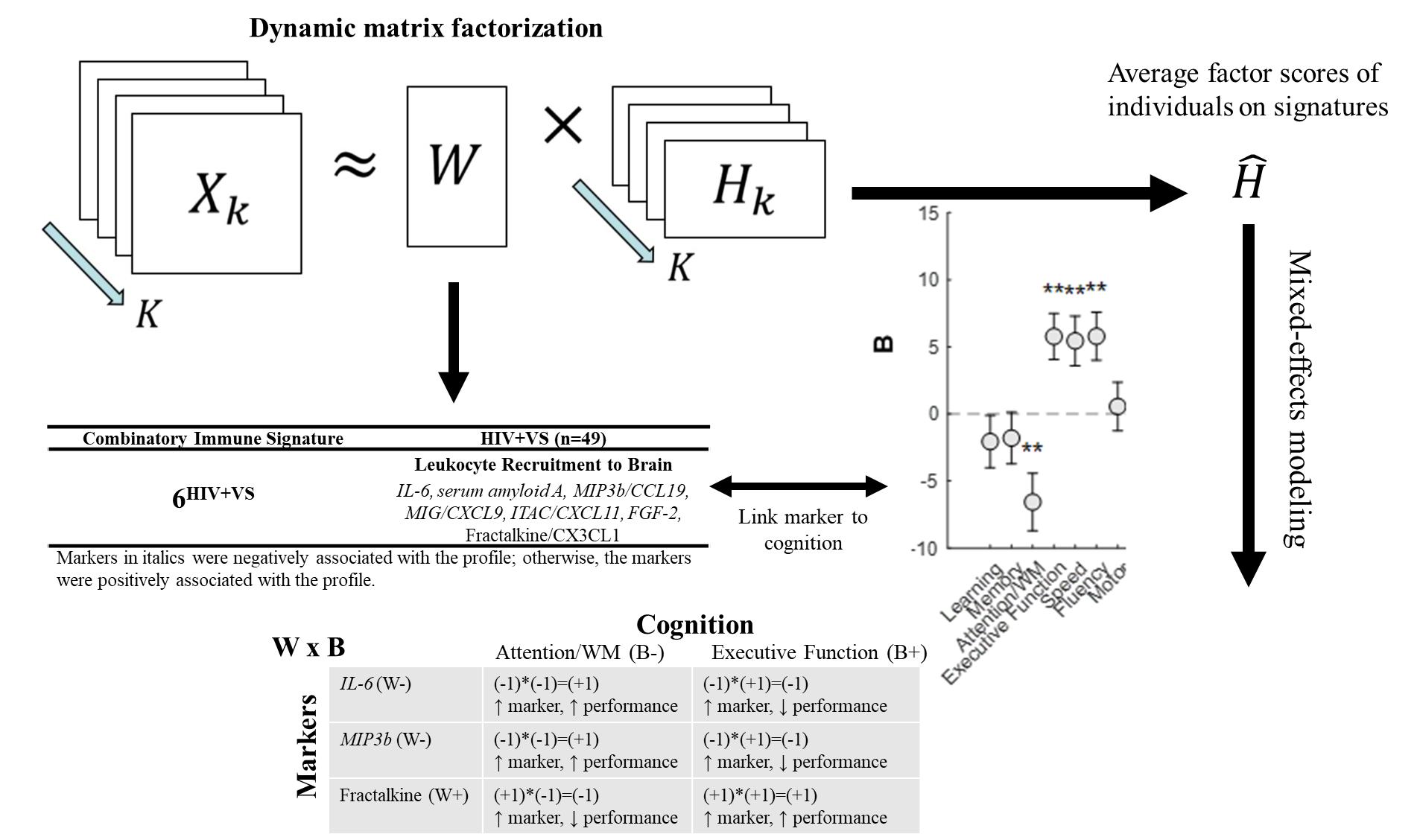

Supplement: Supplementary file 2 [file Data_Sheet_2.docx]
