# Supplementary material for: Early Inflammatory Signatures Predict Subsequent Cognition in Long-Term Virally Suppressed Women With HIV
Source: Front Integr Neurosci. 2020 Apr 24;14:20. doi: 10.3389/fnint.2020.00020 (PMC7193823; doi:10.3389/fnint.2020.00020)
Supplement: Supplementary file 3 [file Table_1.docx]

Supplementary Tables

# Supplementary Table 1.

Demographic, behavioral, and clinical characteristics for the overall sample (n=105) and for the subset with cognitive data (n=83).

| Variable | Overall sample (n=105)  n (%) | Subset with cognitive data (n=83)  n (%) | p-value |
| --- | --- | --- | --- |
| Age, M (SD) | 38.0 (8.9) | 38.8 (9.2) | 0.72 |
| Education  < High school  High school  > High school | 29 (28)  43 (41)  33 (31) | 25 (30)  28(34)  30 (36) | 0.59 |
| Race/ethnicity  Black, non-Hispanic  Hispanic  White  Other | 52 (50)  39 (37)  12 (12)  2 (2) | 50 (60)  21 (25)  10 (12)  1 (2) | 0.38 |
| Annual household income <12,000/year | 47 (46) | 40 (49) | 0.61 |
| Currently employed | 38 (36) | 32 (39) | 0.74 |
| Elevated depressive symptoms^†^ | 36 (35) | 28 (34) | 0.95 |
| Currently smoking | 39 (37) | 35 (42) | 0.48 |
| Recent use |  |  |  |
| Heavy alcohol | 6 (6) | 5 (6) | 0.93 |
| Marijuana | 13 (34) | 13 (34) | 0.99 |
| Crack, cocaine, and/or heroin use | 7 (7) | 6 (7) | 0.88 |
| Hepatitis C RNA positive | 26 (26) | 22 (27) | 0.84 |
| Body mass index | 28.8(6.5) | 28.8(6.8) | 0.91 |
| Hypertension | 24(23) | 22 (27) | 0.56 |
| Diabetes | 9 (9) | 7 (8) | 0.97 |

Note. ^†^CES-D=Center for Epidemiological Studies Depression scale >16 cutoff; current, refers to within the past week; recent, refers to within 6 months of the most recent WIHS visit; heavy alcohol use reflects >7 drinks/week or >4 drinks in one sitting; Variables reported as n (%) were analyzed with Chi-square tests. Variables reported as M (SD) were analyzed with independent t-tests.

# Supplemental Table 2.

Analyte tested, out of range (OOR) values and high and low standards.

# Supplemental Table 3

Descriptive statistics of analytes tested prior to transformation at the first visit.


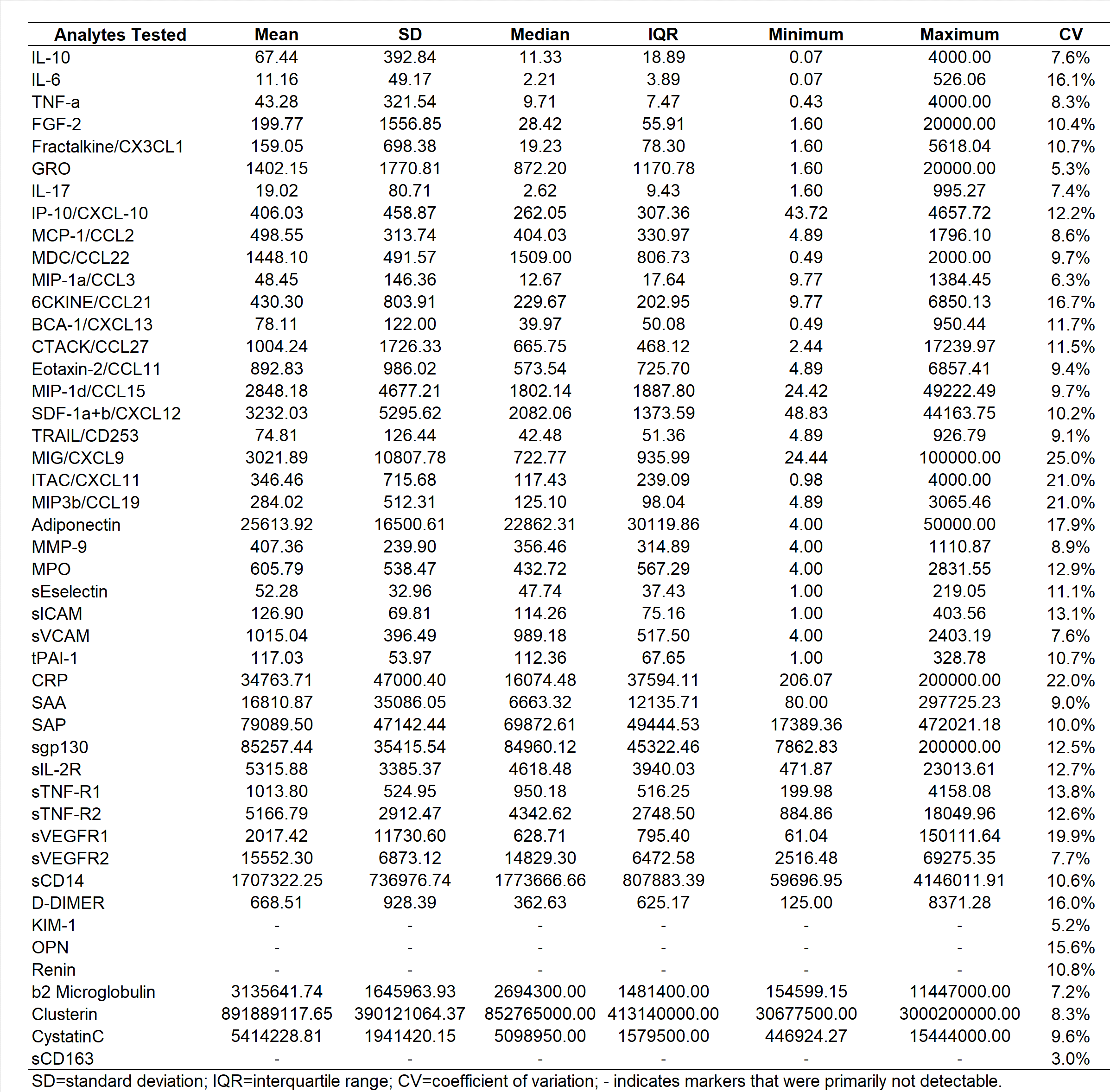


**
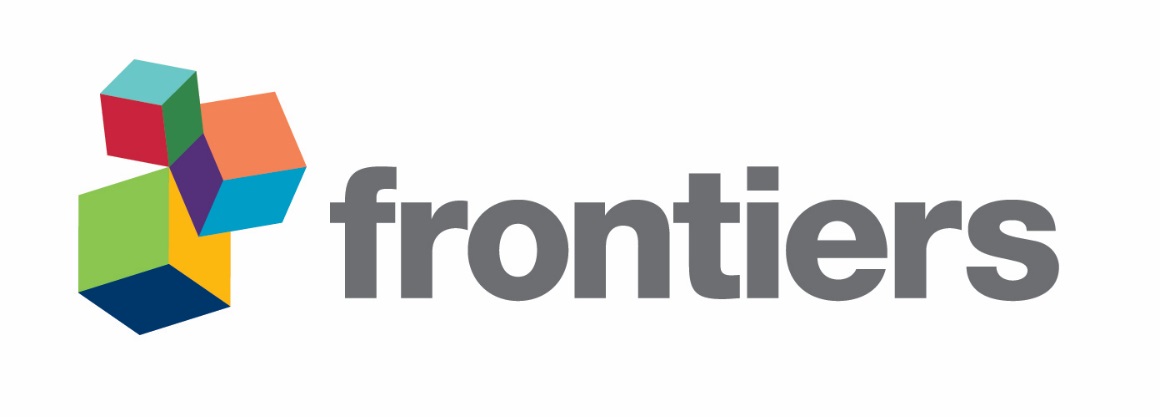
**
